# Deeply conserved synteny resolves early events in vertebrate evolution

**DOI:** 10.1038/s41559-020-1156-z

**Published:** 2020-04-20

**Authors:** Oleg Simakov, Ferdinand Marlétaz, Jia-Xing Yue, Brendan O’Connell, Jerry Jenkins, Alexander Brandt, Robert Calef, Che-Huang Tung, Tzu-Kai Huang, Jeremy Schmutz, Nori Satoh, Jr-Kai Yu, Nicholas H. Putnam, Richard E. Green, Daniel S. Rokhsar

**Affiliations:** 1 Molecular Genetics Unit, Okinawa Institute of Science and Technology Graduate University, Okinawa, Japan; 2 Department of Neuroscience and Developmental Biology, University of Vienna, Vienna, Austria; 3 Université Côte d’Azur, CNRS, INSERM, IRCAN, Nice, France; 4 Department of Biomolecular Engineering, University of California, Santa Cruz, Santa Cruz, CA USA; 5 HudsonAlpha Institute for Biotechnology, Huntsville, AL USA; 6 Department of Molecular and Cell Biology, University of California, Berkeley, Berkeley, CA USA; 7 Dovetail Genomics, Scotts Valley, CA USA; 8 Institute of Cellular and Organismic Biology, Academia Sinica, Taipei, Taiwan; 9 Marine Genomics Unit, Okinawa Institute of Science and Technology Graduate University, Okinawa, Japan; 10 Chan Zuckerberg Biohub, San Francisco, CA USA; 11 Present Address: Centre for Life’s Origins and Evolution, Department of Genetics, Evolution and Environment, University College London, London, UK; 12 Present Address: State Key Laboratory of Oncology in South China, Collaborative Innovation Center for Cancer Medicine, Sun Yat-sen University Cancer Center, Guangzhou, China

**Keywords:** Comparative genomics, Evolution

## Abstract

Although it is widely believed that early vertebrate evolution was shaped by ancient whole-genome duplications, the number, timing and mechanism of these events remain elusive. Here, we infer the history of vertebrates through genomic comparisons with a new chromosome-scale sequence of the invertebrate chordate amphioxus. We show how the karyotypes of amphioxus and diverse vertebrates are derived from 17 ancestral chordate linkage groups (and 19 ancestral bilaterian groups) by fusion, rearrangement and duplication. We resolve two distinct ancient duplications based on patterns of chromosomal conserved synteny. All extant vertebrates share the first duplication, which occurred in the mid/late Cambrian by autotetraploidization (that is, direct genome doubling). In contrast, the second duplication is found only in jawed vertebrates and occurred in the mid–late Ordovician by allotetraploidization (that is, genome duplication following interspecific hybridization) from two now-extinct progenitors. This complex genomic history parallels the diversification of vertebrate lineages in the fossil record.

## Abstract (web summary)

Genomic comparisons with a new amphioxus chromosome-scale genome assembly reveal details of the early evolution of vertebrate genomes.

## Main

In the 1970s, Ohno^1^ proposed that vertebrates arose through a process involving one or more genome-wide duplications. This hypothesis received early support from the discovery of multiple vertebrate Hox clusters compared with one invertebrate cluster^2^ and the finding of numerous vertebrate gene families with members distributed across multiple chromosomes^3,4^. Further evidence came from the discovery of paralogous (that is, duplicated) blocks of linked genes on multiple chromosomes within the human genome^5–8^, culminating in the discovery of widespread quadruply conserved synteny of the human genome^9,10^. These studies support the so-called ‘2R’ scenario of two rounds of whole-genome duplication during vertebrate evolution.

However, the number, timing and mechanism of these duplication events are still debated^3,10–14^. Alternatives to the 2R hypothesis include the recent proposal of a single whole-genome duplication with “additional large paralogy regions being the product of rare segmental duplications occurring both before and after”, based on comparative analyses of the sea lamprey genome^13,15^. Others have suggested a series of large segmental duplications without any genome-wide events^16,17^, although this is a minority view. Contributing to this uncertainty are discrepancies in the inferred chromosomal organization of the proto-vertebrate ancestor. By analysing gene linkages within and among selected bony vertebrate genomes (Euteleostomi), some authors have suggested the existence of 10–13 proto-vertebrate (that is, before any duplications) chromosomes^13,15,18–20^, although other studies^10,14,21^ have inferred 17 ancestral chromosomes.

## Results and discussion

### Amphioxus chromosomes reflect ancestral chordate linkages

**Extended Data Fig. 1:**
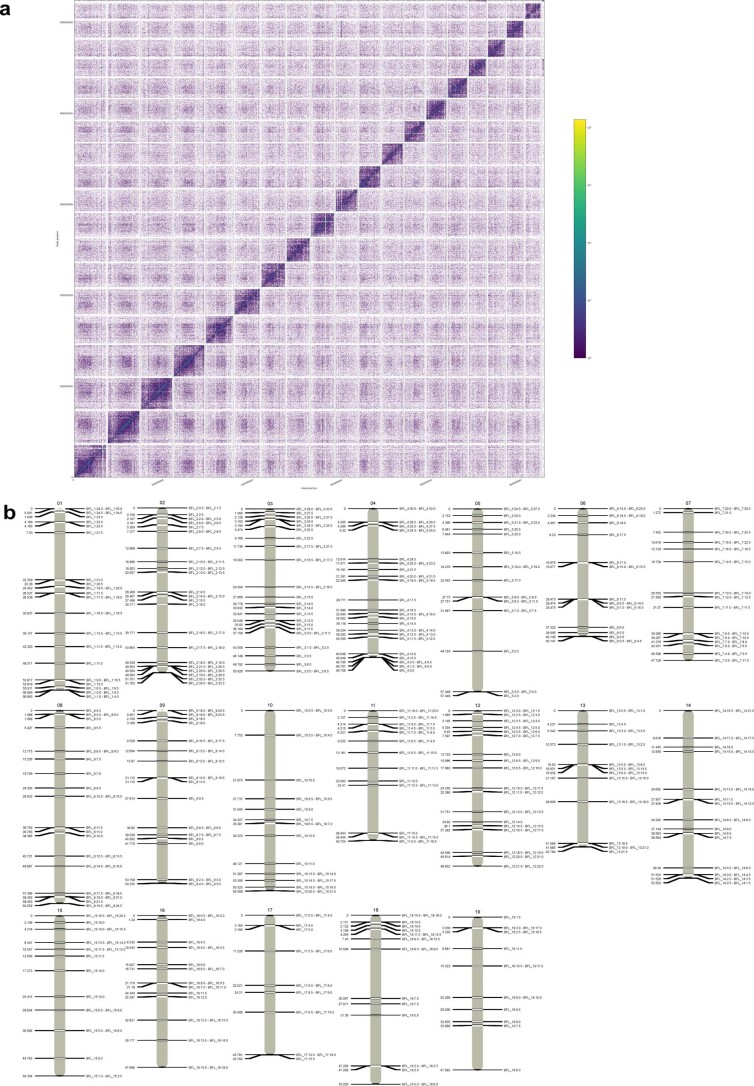
Chromatin and genetic maps of amphioxus genome. (**a**): Chromatin conformation capture contact map for amphioxus genome assembly. Density of read-pairs representing three-dimensional chromatin contacts are shown as a heat map. (**b**): Maternal meiotic linkage map of amphioxus from a 96 progeny F1 cross. Markers represent phased 500 kb windows of the chromosomal assembly; consecutive windows are combined when there is no evidence for recombination in the genotyped progeny. Amphioxus linkage groups and the 19 longest assembled scaffolds are in 1:1 correspondence, confirming the Hi-C-based chromosome-scale assembly. (See Supplementary Note 4.).

As an invertebrate chordate whose lineage diverged before the emergence of vertebrates, amphioxus species have often served as a proxy for the ancestral proto-vertebrate condition^22^, and provide a critical outgroup for analysing vertebrate-specific gene duplications^2–4,10^ and the evolution of vertebrate gene regulation^23^. To robustly infer the proto-vertebrate karyotype and the genomic changes that accompanied the invertebrate-to-vertebrate transition, we produced a chromosome-scale genome assembly of amphioxus (the Florida lancelet *Branchiostoma floridae*). We combined existing shotgun data^10^ with new in vitro^24^ and in vivo^25^ chromatin conformation capture sequences that enable megabase-scale scaffolds to be accurately linked together to reconstruct chromosomes^24,25^ (Methods, Supplementary Notes 1 and 2 and Extended Data Fig. 1a). The resulting chromosome-scale assembly of *B. floridae* represents a substantial improvement over the original draft genome sequence, which achieved only megabase-scale scaffolds^10^, and megabase-scale assemblies of other amphioxus species^23,26^. Our assembly assigns 94.5% of genes to the 19 *B. floridae* chromosomes BFL1–19. We validated the chromosome-scale accuracy of the new *B. floridae* assembly by generating a dense meiotic linkage map made from the F1 progeny of two wild parents (Supplementary Note 3 and Extended Data Fig. 1b)^10,22^.

**Fig. 1:**
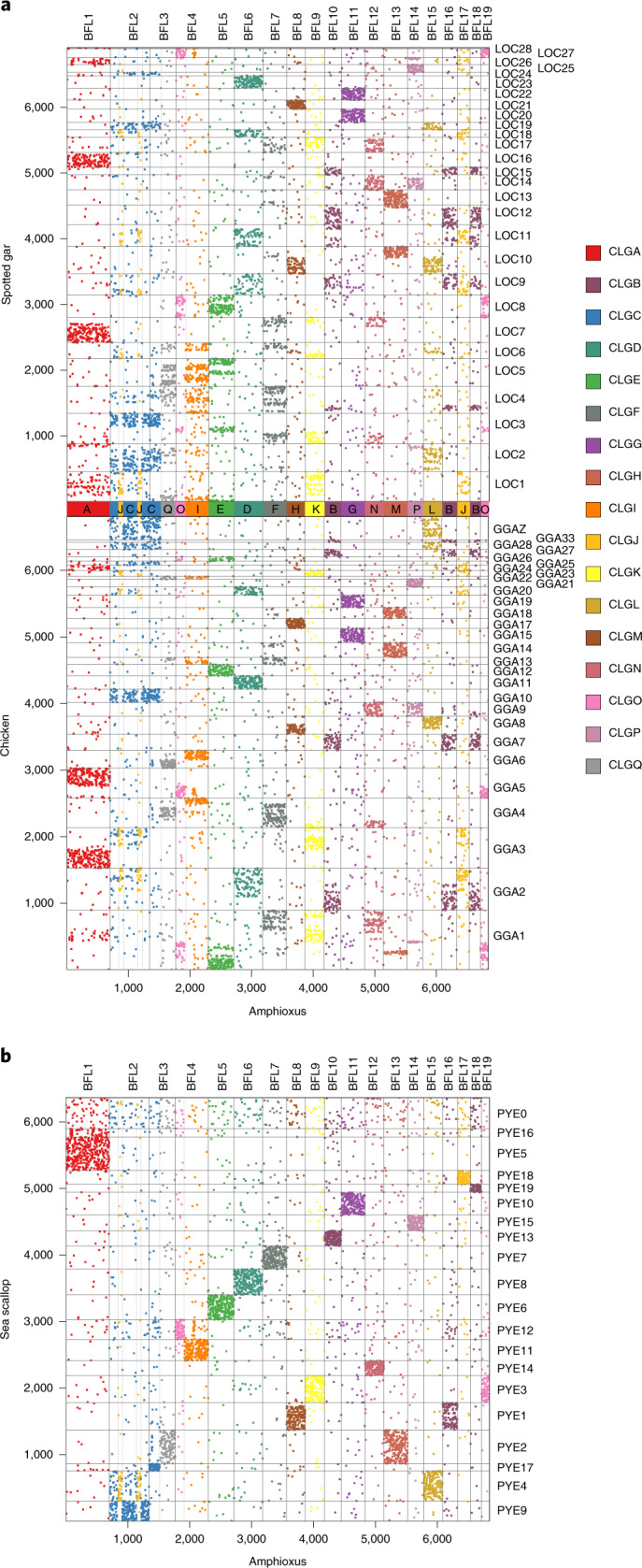
Conserved syntenies between amphioxus and various species. **a**, Oxford dot plot of orthologous genes between amphioxus and two representative bony vertebrates: spotted gar (*Lepisosteus oculatus*; top) and chicken (*Gallus gallus*; bottom). The axes show the index of 6,843 orthologous gene families anchored by mutual best hits from gar, chick, frog and human to amphioxus, with chromosome boundaries indicated. Dashed vertical lines show the location of synteny breakpoints for amphioxus that are consistent in comparisons with other vertebrate (Extended Data Figs. 2 and 3) and invertebrate genomes (see **b**; Extended Data Fig. 4). Genes are coloured according to this partitioning, defining 17 ancestral CLGs, with labels shown to the right. **b**, Mutual best-hit dot plot of amphioxus versus scallop, using the same colouring as in **a**. Syntenic discontinuities in amphioxus (indicated by the dashed lines) are consistent in the scallop. Note that CLGB (dark purple) is distributed across three pairs of homologous chromosomes, implying that this CLG existed as three distinct linkage groups in the scallop–amphioxus common ancestor.

**Extended Data Fig. 2:**
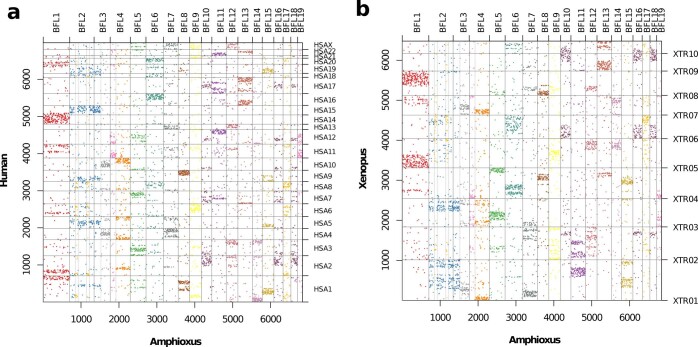
Dot-plots showing conserved syntenies between amphioxus and human and frog. Dots represent mutual best hits between amphioxus and frog (*Xenopus tropicalis*, XTR) and human (*Homo sapiens*, HSA). Only mutual-best-hits involving the 6,843 genes of Fig. 1 are considered. (These gene families are anchored by mutual best hits between the four jawed vertebrate representatives and amphioxus.) Genes are colored based on their CLG membership as in main Fig. 1. Horizontal and vertical solid lines represent chromosome boundaries; vertical dashed lines represent inferred synteny breakpoints in amphioxus as in Fig. 1 (Methods).

**Extended Data Fig. 3:**
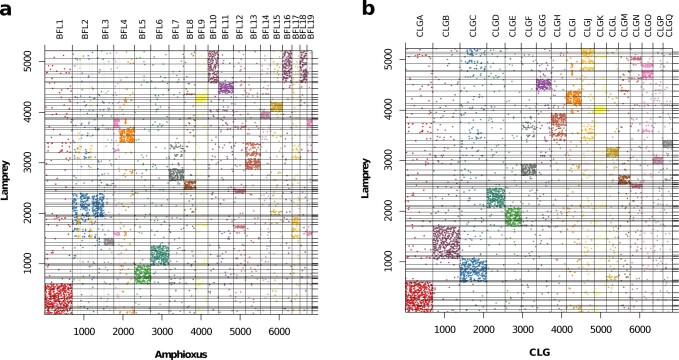
Dot-plots showing conserved syntenies between lamprey and amphioxus. Dots represent mutual best hits between the (germline) genome of the sea lamprey (*Petromyzon marinus*) and amphioxus. Genes are colored based on their CLG assignment, and lamprey chromosomes are sorted according to their CLG content. Panel a shows these distribution of orthologous genes vs. amphioxus chromosomes, revealing the same discontinuities (vertical dashed lines) in amphioxus-lamprey synteny as found for amphioxus-bony vertebrate comparisons shown in Fig. 1 and Extended Data Fig. 2. Panel b shows these same orthologous gene pairs versus CLGs.

**Extended Data Fig. 4:**
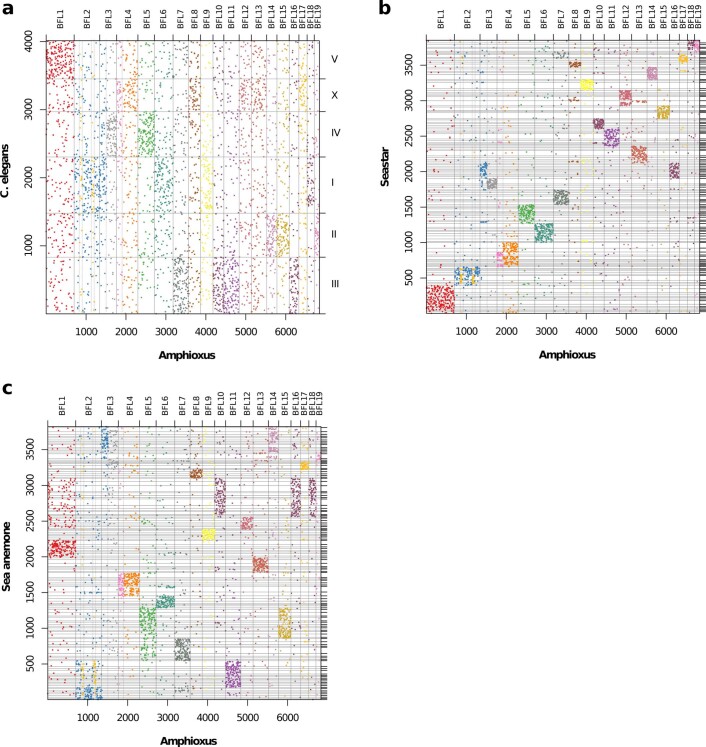
Dot-plots showing conserved syntenies between amphioxus and selected invertebrates. Dots represent mutual best hits between amphioxus and the genomes of the Crown Of Thorns sea star Acanthaster planci, the soil nematode Caenorhabditis elegans, and the starlet sea anemone Nematostella vectensis. The N. vectensis and A. planci genomes are not yet assembled into chromosomes, and only scaffolds containing 20 or more genes (counting only mutual-best-hit vs. amphioxus) are shown. Scaffolds are sorted based on clustering using similarity of their CLG content. Vertical dashed lines are as shown in and Fig. 1, with the same CLG-based coloring, showing that the partitioning of amphioxus found using jawed vertebrates is also consistent with diverse invertebrates, and that sea star and sea anemone scaffolds can be grouped according to conserved synteny with amphioxus. C. elegans chromosomes arose by fusion, translocation, and mixing of the ancestral bilaterian units that are still retained in amphioxus.

To examine the conservation of syntenic relationships, we constructed Oxford dot plots comparing the chromosomal positions of orthologous genes between genomes of amphioxus and multiple vertebrates (Fig. 1a and Extended Data Figs. 2 and 3) and invertebrates (Fig. 1b and Extended Data Fig. 4). These plots clearly display dense rectangular blocks of dots that represent units of deeply conserved synteny. Here, we use the original meaning of ‘synteny’^27^ to represent physical linkage without regard to gene order. The uniform distribution of orthologues within these blocks implies that while physical linkage is conserved, gene order within syntenic units has become largely scrambled since the amphioxus and other lineages diverged from each other more than half a billion years ago. This gene order scrambling is the result of accumulated inversions and other intra-chromosomal rearrangements over time, as observed between *Drosophila* species across increasing evolutionary distances^28,29^.

Comparison of the chromosomes of amphioxus and the scallop *Patinopecten yessoensis* (chromosome code: PYE)^30^—the most complete chromosomal assembly of a marine invertebrate until amphioxus, albeit with only 80% of genes assigned to linkage groups—shows the remarkable stability of bilaterian chromosomes. Many amphioxus and scallop are in 1:1 correspondence, and others are related by the limited rearrangements described below (Fig. 1b). This observation directly confirms the deep conservation of synteny we previously hypothesized based on networks of conserved linkages observed among draft genomes of diverse invertebrates^21,30–33^ (see Extended Data Fig. 4).

We identified 17 distinct patterns of conserved amphioxus–vertebrate synteny (Supplementary Note 4). Each pattern represents a group of genes whose linkage has been preserved since the divergence of the vertebrate and cephalochordate lineages, and is identified with an ancestral chordate linkage group (CLG). Each CLG is assigned a letter (A–Q, in decreasing order of number of genes) and, for ease of representation, colour, consistently used throughout. Vertical dashed lines show sharp boundaries between segments of different CLG ancestry in amphioxus; these are consistent in all comparisons among both vertebrates and invertebrates (Fig. 1 and Extended Data Figs. 2–4). The CLGs defined here by amphioxus–vertebrate comparisons also are found as intact units in the scallop (Fig. 1b and Supplementary Note 4), which implies that they represent even more ancient bilaterian or metazoan conserved syntenic units.

The amphioxus karyotype (*n* = 19) is derived from the 17 ancestral CLGs through a handful of large-scale rearrangements. Twelve amphioxus chromosomes (BFL1, 5–9, 11–15 and 17) are in 1:1 correspondence with CLGs and are therefore direct descendants of these ancestral units, albeit with extensive internal gene order rearrangement (Fig. 1). The remaining amphioxus chromosomes were formed through a small number of translocations between ancestral units. Three of the longer amphioxus chromosomes (BFL2, 3 and 4) are each derived from pairs of CLGs, with sharp boundaries between distinct patterns of conserved synteny across the chicken (chromosome code: GGA), gar (chromosome code: LOC), human (chromosome code: HSA), frog (chromosome code: XTR), sea lamprey and scallop (vertical dashed lines Fig. 1 and Extended Data Figs. 2 and 3). BFL2 exhibits an alternating block pattern of CLGJ and C ancestry that plausibly arose through a pair of overlapping inversions that occurred after a translocation involving a common ancestor with BFL17, which shares CLGJ ancestry with BFL2. Since the CLG boundaries remain sharp in amphioxus, these rearrangements must have occurred recently on the time scale of gene order scrambling. Alternately, sharp boundaries could represent current or historical centromeres, which interfere with mixing across arms.

The trio of amphioxus chromosomes BFL10, 16 and 18 together correspond to the ancestral CLGB. However, each of these chromosomes is associated with a different scallop chromosome (Fig. 1). It follows that these each represent a conserved ancestral bilaterian unit, implying at least 19 basic elements in the bilaterian ancestor. However, since orthologues of BFL10, 16 and 18 always occur mixed together in bony vertebrates and lamprey, we infer that these three elements fused before the origin of vertebrates, and for the purposes of our vertebrate-centric analysis treat them as a single CLG unit below. The stability of the amphioxus karyotype relative to these ancestral units is consistent with the megabase-scale conserved synteny (Supplementary Note 6) and minor karyotypic differences observed between *Branchiostoma* species^34^. The amphioxus genome shows no evidence of large-scale duplication.

### Deep ancestry of vertebrate chromosomes

**Fig. 2:**
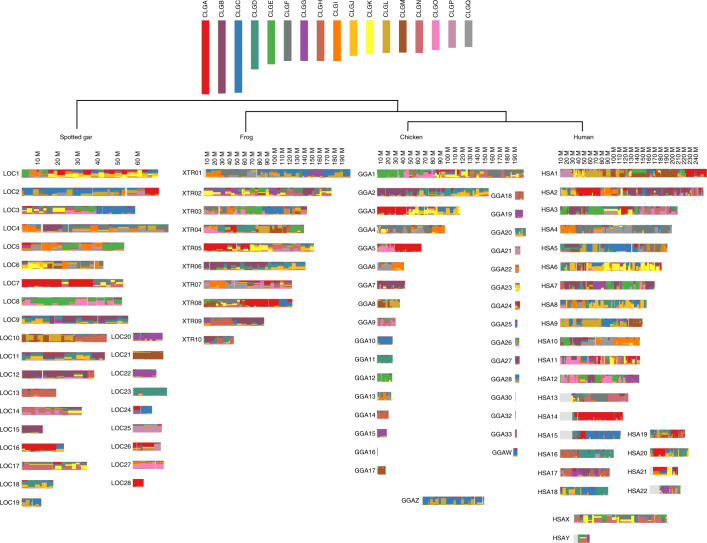
Contributions of the 17 ancestral CLGs to contemporary vertebrate genomes. The CLG ancestries of four jawed vertebrate genomes are shown by the local fraction of genes that are derived from each CLG, in windows of approximately 20 genes (see Methods). Note that, in contrast with Fig. 1, the chromosomal position is shown as physical coordinates (that is, base pairs), so area is not proportional to gene number. Colours are the same as in Fig. 1. The statistical significance of the associations between CLGs and vertebrate chromosomes is reported in Fig. 3.

With the 17 ancestral chordate linkage units in hand, we can infer the sequence of genomic events that produced modern vertebrates. From Fig. 1, we can determine the distribution of CLG ancestry across the bony vertebrate genomes as shown in Fig. 2. Vertebrate chromosomes generally comprise one or more large blocks that are either: (1) descendants of single CLGs (dominated by a single colour; for example, the block of pure CLGA ancestry of the left arm of GGA3 and right arm of GGA5); or (2) mixtures of two or three CLGs formed by fusion and subsequent rearrangement (multiple overlapping or interleaved colours; for example, the CLGC and CLGL ancestry of GGAZ). Since chicken, spotted gar, and sea lamprey show fewer chromosomal rearrangements than human and frog, we focus on these genomes in the main text as more closely reflecting ancestral vertebrate genome organization.

**Fig. 3:**
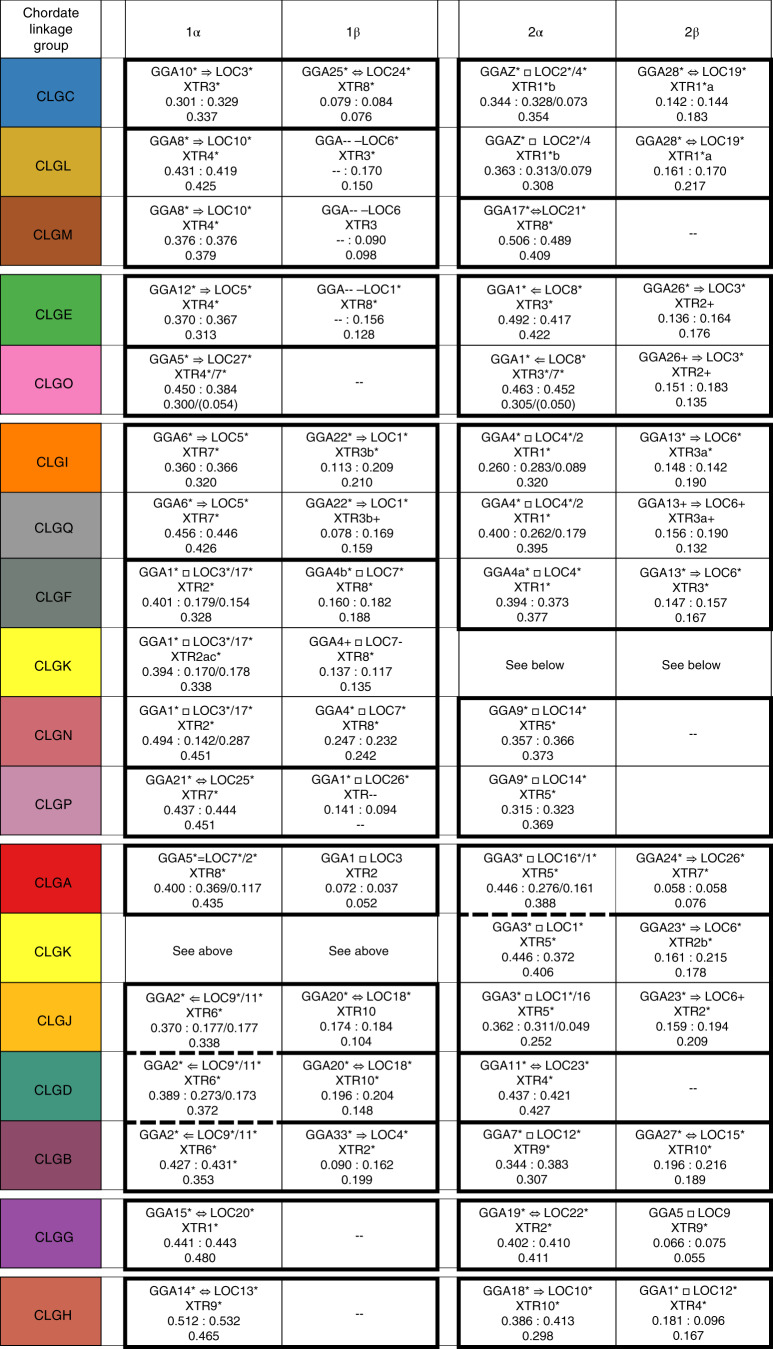
Organization of bony vertebrate chromosomes after 2R. The majority of CLGs have four copies in bony vertebrates; the remainder have three. Organizing these copies by chromosome fusion (solid rectangles joining cells) and gene retention (numbers in cells) shows that chicken, spotted gar and frog chromosomes can be sorted into ‘α–β’ pairs that share the same patterns of CLG fusion, and these pairs themselves form ‘1–2’ pairs. Bold dashed lines separating CLGA-2α and CLGB-1α from their fusions with other CLGs indicate either fusions in the α-lineage or fissions in β. Due to this ambiguity, the β pairings in these two rows are arbitrary. Similarly, the β copies for CLGG and CLGH are arbitrarily assigned to 2. In several cases (for example, CLGO) two distinct copies are found on the same chromosome of one species; these are indicated as a and b. Arrows imply that the entire source chromosome is orthologous to the target; double-headed arrows indicate reciprocal orthology; boxes indicate that segments of the chromosomes are orthologous; -- indicates undetected enrichment. The significance of associations between CLG and jawed vertebrate chromosomes was determined as described in Methods. Significance determined using 50-gene windows (*P* < 0.01) is indicated by an asterisk. Significance determined using 50-gene windows (*P* < 0.05), 100-gene windows (*P* < 0.01) and/or at the whole-chromosome level (*P* < 0.05) is determined by a plus sign. All *P* values were Bonferroni corrected.

We find that micro-chromosomes of the chicken, gar and lamprey (defined in ref. ^35^ as chromosomes shorter than 15 megabases) typically descend from single CLGs (Supplementary Note 6). Furthermore, micro-chromosomes of the chicken and spotted gar are often orthologous^34^ (Fig. 3). For example, GGA28 and LOC19 both descend from CLGC and are orthologous (their 1:1 relationship is indicated by the double-headed arrow symbol in Fig. 3). These observations not only imply that such acrocentric micro-chromosomes were present in the common bony vertebrate ancestor^19,36^, but also that they are relicts of even more ancient micro-chromosomes of the last common chordate ancestor, many of which are preserved in amphioxus. Remarkably, all CLGs appear at least once in unmixed form in the sea lamprey (see Extended Data Fig. 3), and nine out of 17 (CLGA, B, C, D, G, H, K, M and P) are also found in their ancestral unfused form in at least one bony vertebrate. This implies that at least one descendant of each original CLG has persisted (albeit with gene loss; see below) since the earliest periods of vertebrate evolution.

In contrast with micro-chromosomes, the longer metacentric macro-chromosomes of bony vertebrates are typically concatenations of segments with either distinct single CLG ancestry or blocks of mixed CLG ancestry (Fig. 2). Sharp boundaries between blocks of differing CLG ancestry represent either translocation boundaries or contemporary or ancient centromeres. The centromere scenario is consistent with the hypothesis that CLGs represent ancient chordate chromosome arms and implies that some metacentric vertebrate chromosomes arose through ancient Robertsonian fusions/translocations^37^. In this sense, the CLGs can be thought of as the vertebrate analogues of the Muller arms of *Drosophila*, which have maintained their integrity during fruit fly evolution despite considerable internal rearrangement^28,29^.

**Fig. 4:**
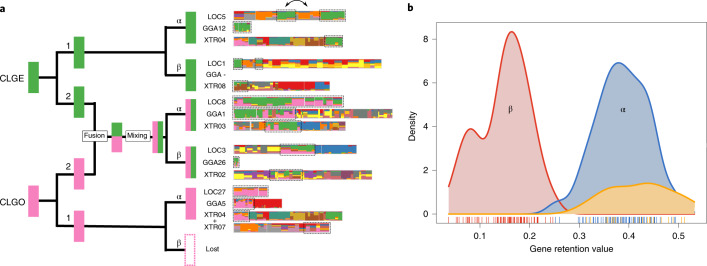
Duplications, fusions and mixing in bony vertebrates. **a**, Right: chromosomal descendants of CLGE (green) and CLGO (pink) are organized into five groups. Each chromosome is represented as in Fig. 2, with corresponding segments outlined by black dotted rectangles. The double-headed arrow indicates probable inversion that separated two CLG blocks. Within each group, segments with the CLGE and/or CLGO ancestry are orthologous among the chicken, gar and frog, and groups are paralogous to each other. Note that the frog chromosome XTR4 has distinct CLGE and CLGO segments with distinct ancestry (see Supplementary Note 6). Left: cladogram showing the most parsimonious evolutionary history leading to these vertebrate chromosomes, starting from CLGE and CLGO ancestors. This includes an early duplication (producing copies labelled 1 and 2), a fusion and subsequent mixing, and then a second duplication (producing copies labelled α and β). The CLGO-1β copy was not found, as indicated by a dashed pink rectangle. CLGE-1β was not found in chicken, as indicated by the dash. CLGO-1α was found split across XTR04 and XTR07, as indicated by the plus sign. **b**, Distribution of gene retention for the α and β segments listed in Fig. 3, with rug plot and kernel density estimator. The upper curves are for α–β pairs, whereas the orange curve is for α segments without β counterparts (presumed lost or possessing limited gene content and therefore undetected).

**Extended Data Fig. 5:**
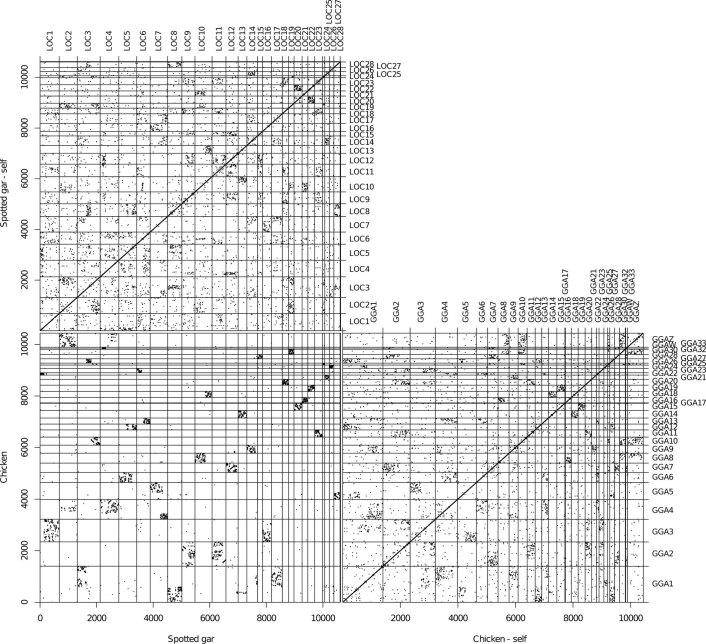
Chicken-spotted gar orthologs and paralogs. “Oxford’ dotpot between chicken (Gallus gallus, GGA) and spotted gar (Lepisosteus oculatus, LOC). Dots in the lower left corner represent mutual best hits between chicken and spotted gar, showing the clear orthologous blocks conserved synteny that allows chicken and spotted gar chromosome segments to be placed in correspondence with each other. Upper left and lower right show intra-genomic non-self best hits that identify paralogous regions within the chicken and spotted gar genome, respectively. Paralogous chromosomal regions share the same chordate linkage group ancestry, but arose through duplication.

Numerous instances of duplication, fusion and mixing can be seen in Figs. 1 and 2, and their pattern across genomes reveals the ancient dynamics of vertebrate chromosomes. Consider, for example, the distribution of genes with CLGE (green) and CLGO (pink) ancestry across bony vertebrate genomes. In Fig. 4a, chicken, gar, and frog chromosomes with E and O ancestry are arranged into five paralogous sets (Extended Data Fig. 5; see also Supplementary Note 6), revealing that these bony vertebrates generally have: (1) a pair of E-only segments (although one of the E-only segments is missing or dispersed and/or not detected in the chicken); (2) one O-only segment; and (3) two segments with mixed CLGE/CLGO ancestry. We interpret these blocks of mixed ancestry as arising from the past fusion of segments of E and O ancestry followed by a series of local rearrangements. The existence of pure E and O segments in the outgroups amphioxus and scallop indicates that pure CLG ancestry was the ancestral state and that these mixtures arose by fusion. This ancestral proto-vertebrate state is further corroborated by the absence of E–O fusions in the lamprey, which implies that the E–O fusion occurred on the bony vertebrate stem after divergence from the lamprey.

The cladogram on the left of Fig. 4a shows the most parsimonious derivation of these bony vertebrate segments from CLGE and CLGO chromosome ancestors. In particular, we note that it is more parsimonious for the two mixed-ancestry segments to arise by duplication after a common ancestral fusion/mixing than for two independent E–O fusion/mixing events to occur. Since all bony vertebrates possess the two fused segments, the duplication must have occurred before the tetrapod–gar divergence. Similarly, the co-existence of E-only, O-only and E–O fused segments implies duplication of E and O before the fusion/mixing event. The descendants of these early duplications are labelled 1 and 2, while the products of the second set of duplications are labelled α and β, in keeping with the notation detailed below. In this scenario, the β copy of O-1 has been lost (shown as a dashed pink rectangle).

Figure 3 extends this logic across vertebrate genomes to reveal a network of ancient duplications, fusions and mixing. Each cell in the table corresponds to the bony vertebrate descendent of an ancestral chordate unit, represented by a trio of orthologous chromosome segments of the chicken, spotted gar and frog (Methods). The cells are arranged according to CLG ancestry (rows), with paralogous copies in different columns. Solid lines enclose conserved linkages among CLGs (that is, juxtapositions or mixtures of CLG ancestry on orthologous vertebrate chromosomes) across bony vertebrates. In the vast majority of cases, segments on different chromosomes that descend from the same CLG arose by ancient duplication, as can be seen by the distribution of paralogous genes within jawed vertebrate genomes^9,10,19,20^ (Extended Data Fig. 5). The alternative situation, in which CLG blocks are split across multiple chromosomes due to past translocations or fissions, is rarer but does occur^10,19^, and can be inferred by parsimony when their orthologues are maintained as a single block in another jawed vertebrate genome, with amphioxus and scallop serving as outgroups. For example, the segments of LOC9 and 11 with CLGB, D and J ancestry are together orthologous to GGA2, and therefore probably arose by translocation after divergence of the gar and tetrapod lineages. Conversely, there are cases where two paralogous blocks with the same CLG ancestry are found on the same frog chromosome; consistent orthology with the gar and chicken allows these blocks to be identified and placed in different cells in Fig. 3.

### Patterns of duplication and fusion in early vertebrate evolution

**Fig. 5:**
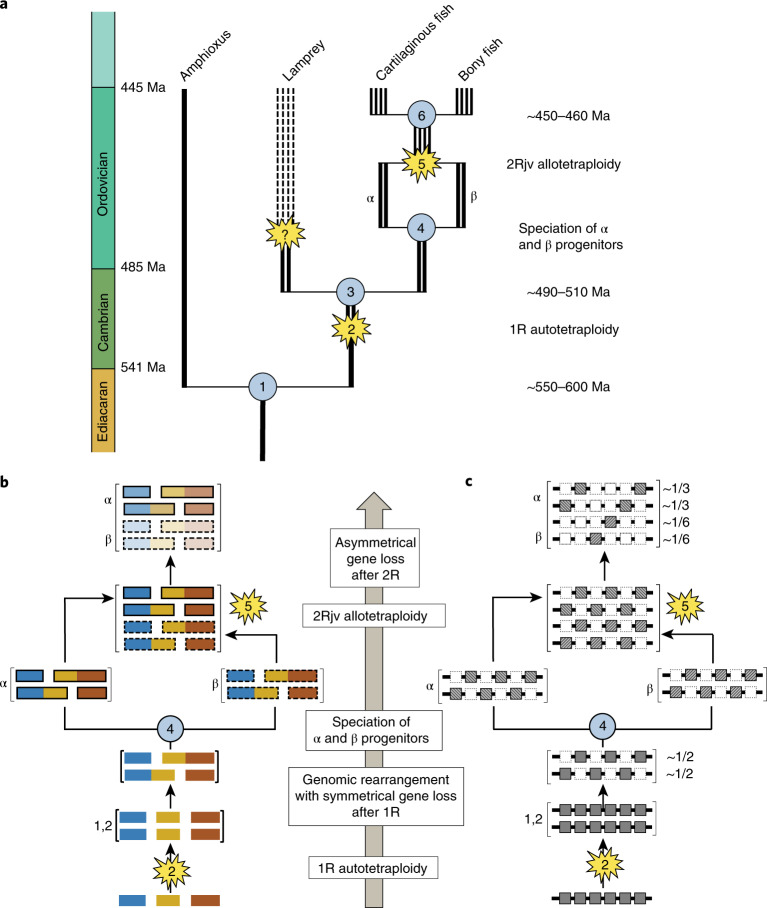
Auto- then allotetraploidy scenario for vertebrate evolution. Schematic of the auto- then allotetraploidy scenario described in the main text. **a**, Each line represents a chromosomal lineage. Single lines represent diploids, paired lines represent tetraploids, and so on, relative to the ancestral chordate chromosome complement. Dashed lines later in the lamprey lineage reflect one or more additional genomic duplications. Labelled nodes: (1) divergence of amphioxus and vertebrates (last common chordate ancestor); (2) 1R autotetraploidy, resulting in genome doubling; (3) divergence of (tetraploid) lamprey and gnathostome progenitor lineages; (4) speciation of palaeotetraploid gnathostome progenitors; (5) 2Rjv allotetraploidy, in which palaeotetraploid gnathostome progenitors hybridize to form the crown gnathostome lineage, which is quadrupled relative to the chordate ancestor; (6) divergence of extant jawed vertebrate lineages. The question mark indicates one or more additional duplication(s) that may have occurred in the lamprey lineage. **b**, Schematic showing the evolution of three ancestral CLGs. Relevant nodes are labelled as in **a**. Bold and dashed boundaries around chromosomes in the α and β lineages, respectively, represent divergences that accumulate in each lineage. Differential shading after node 5 indicates subsequent gene loss. **c**, Schematic of the evolutionary history of six linked chordate genes through vertebrate duplications. Gene loss is symmetrical after autotetraploidy (node 2) but asymmetrical after allotetraploidy (node 5). For simplicity in this diagram, gene order changes are not shown. Cross-hatching indicates independent differentiation in α and β lineages. Empty dashed boxes follow the fate of lost genes.

**Extended Data Fig. 6:**
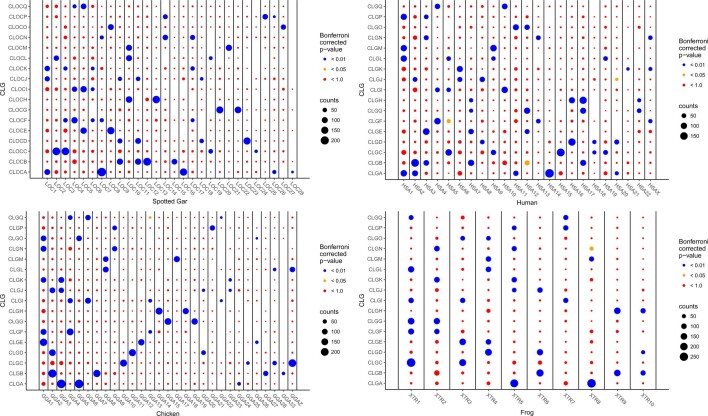
Oxford grid between bony vertebrate chromosomes and chordate linkage groups (CLGs). Circles represent the number of orthologous genes between human (Homo sapiens, HSA), chicken (Gallus gallus, GGA), frog (Xenopus tropicalis, XTR), and spotted gar (Lepisosteus oculatus, LOC) and the seventeen chordate linkage groups (CLGs) described in the text. Orthology is operationally defined by mutual best hits, restricted to 6,843 gene families anchored by mutual best hits of the four jawed vertebrates to amphioxus. The area of each circle is proportional to the number of orthologous genes for each chromosome-CLG pair, and the color indicates the significance of the association relative to a null model in which the position of the orthologous genes are randomly shuffled (Methods).

**Extended Data Fig. 7:**
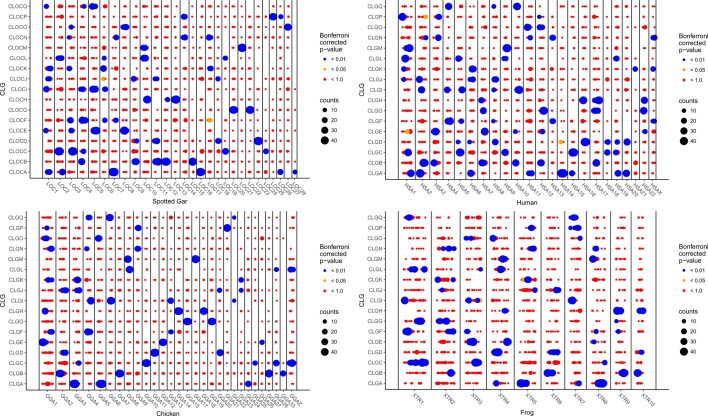
Oxford grid showing associations between 50 gene segments of bony vertebrate chromosomes and chordate linkage groups (CLGs). Given the evident localization of orthologs along bony vertebrate chromosomes shown in the ‘Oxford’ dotplots of Fig. 1 and Extended Data Figs. 2 and 3, we assessed the significance of associations between sub-chromosomal regions and the chordate linkage groups. Each vertebrate chromosome was divided into overlapping 50 gene windows (offset by 25 genes). Only 6,843 genes with amphioxus-bony vertebrate mutual best hits are used. Circle areas are proportional to the number of orthologous genes for each chromosome-CLG pair, and the color indicates the significance of the association relative to a null model in which the position of the orthologous genes are randomly shuffled. Comparing Extended Data Figs. 6 and 7 shows additional significant associations that are missed based on whole chromosome analyses.

**Extended Data Fig. 8:**
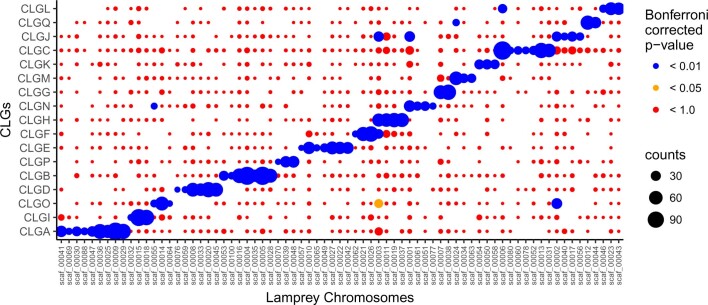
Oxford grid between sea lamprey germline chromosomes and chordate linkage groups (CLGs). Circles show the number of orthologous genes between germline chromosomes of sea lamprey (Petromyzon marinus, PMA, denoted scaff_XXXXX following Smith et al.) and the seventeen CLGs described in the main text. As in Extended Data Fig. 6 the area of each circle is proportional to the number of orthologous genes for each chromosome-CLG pair, and the color indicates the significance of the association relative to a null model in which the position of the orthologous genes are randomly shuffled. Sea lamprey chromosomes and CLGs are both sorted to exhibit the striking correspondence between them. Each of the 17 CLGs is represented by at least one lamprey chromosome, and typically 6-8 lamprey chromosomes are associated with teach CLG. Compare Fig. 4b of Smith and Keinath 2015, which compares lamprey chromosomes to ‘putative ancestral linkage groups’ derived by Putnam 2008 through clustering of amphioxus scaffolds These putative ancestral linkage groups are in 1:1 correspondence with the CLGs shown here to be represented as large chromosomal segments of amphioxus.

The hidden structure of vertebrate chromosomes revealed in Fig. 3 exhibits several remarkable patterns that: (1) imply two distinct tetraploidizations in the history of bony vertebrates; and (2) constrain the mechanisms and timing of those duplications. These observations lead us to propose a novel scenario for vertebrate palaeopolyploidy that is shown in Fig. 5. Since our inferences are derived from discrete patterns of conserved macro-synteny involving significant (see *P* values in Fig. 3 and Extended Data Figs. 6–8) conserved linkages of dozens to hundreds of genes, they are robust to phylogenetic artefacts of modelling sequence evolution.

First, the majority of CLGs (ten out of 17) are found in four descendent copies in bony vertebrate genomes (Fig. 3); the remainder are found in three copies. This pattern supports the 2R hypothesis if we allow for secondary chromosome loss via ancient aneuploidy of initially quadruply redundant copies. We note that gene loss, which can be extensive after genome duplication (see below), also reduces our power to detect statistically significant segments of conserved synteny, especially for the CLGs that contain fewer genes, so that some empty cells may be due to our inability to confidently detect them. Extensive gene loss after polyploidy also makes subsequent aneuploidy less disruptive and therefore more plausible. Figure 3 is consistent with previous studies^9,10,14,19,20^ that find approximately fourfold jawed vertebrate paralogy, as expected for a 2R scenario. Our findings are notably more extensive than the relatively limited jawed vertebrate paralogies recently reported in refs. ^13,15^, which led these authors to propose only a single whole-genome duplication during vertebrate evolution.

Second, Fig. 3 shows that bony vertebrate chromosomes with shared combinations of CLG ancestry generally appear in paralogous α–β pairs. Each α–β pair exhibits the same CLG linkages in the spotted gar, chick and frog, as shown by solid lines surrounding adjacent α and β cells in Fig. 3. (The two exceptions—A2/KJ2 and B1/DJ1—are shown with dashed lines in Fig. 3. These differences between α and β are plausibly accounted for by fusion in the α lineage after divergence from β.) These linked CLG combinations reflect ancient fusions or translocations^37^, followed by more or less extensive mixing. By parsimony, we infer that the CLG fusions shared by paralogous α and β copies predate the genome-wide duplication that produced these α–β pairs, as shown in Fig. 4a. The α–β duplication also evidently preceded the divergence of bony vertebrate lineages, since α and β copies are found in both spotted gar and tetrapods. We reject the alternative scenario in which β copies independently fused in the same pattern as α copies (*P* < 10^−10^; Methods).

Third, each CLG generally participates in two α–β pairs of bony vertebrate segments (which we arbitrarily labelled 1 and 2 in Fig. 3**)**. The genomic duplication that produced the 1–2 pairs therefore must have preceded the α–β duplication. We also infer from Fig. 3 that most CLG fusions in bony vertebrates occurred between the 1–2 and α–β duplications. CLGG and CLGH have evidently not fused; CLGI and CLGQ were either anciently joined before the 1–2 duplication, perhaps as arms of a single metacentric chromosome, or fused independently after it. This behaviour extends the scenario shown in Fig. 4a.

Finally, we can place the divergence of lamprey and bony vertebrate lineages relative to these duplications by noting that almost all of the CLG fusions observed in bony vertebrates are absent in the sea lamprey; indeed, most lamprey chromosomes are directly descended from single CLGs (Extended Data Figs. 3 and 8). This observation further corroborates the ancestral proto-vertebrate nature of these 17 syntenic units, and implies that most of the ancient bony vertebrate-specific fusions shown in Fig. 3 occurred after the divergence of the lamprey lineage. The exception is the C–L fusion found in a single lamprey chromosome (scaf_0006), which has a paralogous α–β pair in bony vertebrates. This observation suggests that either the C–L fusion occurred before the divergence of the lamprey and jawed vertebrate lineages, or convergent C–L fusions occurred in the two lineages. However, since unfused copies of CLGC and CLGL also exist in the lamprey, we infer that the 1–2 duplication occurred before the split between lamprey and jawed vertebrates. This duplication shared by lamprey and jawed vertebrates corresponds to the single event identified by Smith et al.^13^. The α–β duplication, however, occurred on the jawed vertebrate lineage after it split from lamprey. The fusions that intervene between the 1–2 and α–β events imply that the two duplications were temporally distinct (Fig. 5).

### Asymmetrical paralogue retention and the mechanism of vertebrate genome duplication

Remarkably, we can infer the mechanisms of the temporally distinct 1–2 and α–β duplications by examining the chromosomal distribution of vertebrate orthologues relative to the unduplicated amphioxus genome. In the aftermath of a genome duplication, most duplicated genes are rapidly lost, so that paralogous segments retain a subset of the original gene complement^38–40^. However, the relative uniformity of gene loss depends on the underlying mechanism of genome duplication^41,42^. Following autotetraploidization (genome doubling within a species), we expect that gene losses should be evenly distributed across (initially homologous) duplicated chromosomes—a symmetry enforced by persistent tetrasomic inheritance immediately following autotetraploidy^43–45^. In contrast, allotetraploidization (genome doubling accompanying interspecific hybridization) is an inherently asymmetrical process that brings together the genomes of two progenitors with distinct epigenetic landscapes, cytonuclear interactions and histories of transposable element activity. Allotetrapoids are therefore expected to show an asymmetrical distribution of gene losses, as observed in palaeo-allotetraploid plants^42^ and frogs^41^.

Each cell of Fig. 3 reports the retention fraction in the chicken, spotted gar and frog relative to the corresponding linkage group of the (unduplicated) chordate ancestor (using amphioxus segments to represent ancestral content; see Methods). Under a model in which (1) all chromosomal copies are equivalent (as expected, for example, for two successive autotetraploidies) and (2) redundancies are completely eliminated by gene loss, we would expect ~25% retention per segment (note that this simple picture neglects the relatively small fraction of genes retained in multiple copies^10^, including well-known genes such as those in the Hox, Wnt and Fox families; see Methods). Instead, we observe a strikingly asymmetrical pattern in which genes are more than twice as likely to be retained on α segments than on paralogous β segments (Fig. 4b, Methods and Supplementary Note 6). This asymmetry implies that the α–β duplication occurred through allotetraploidy. In contrast, no asymmetry is observed between 1–2 duplicates (combining the corresponding α–β descendants), which suggests that this earlier duplication occurred by autotetraploidy. Our full scenario is shown in Fig. 5.

The distribution of gene retention fractions across spotted gar, chicken and frog (Fig. 4b) is clearly bimodal (unimodality rejected by Hartigan’s dip test; *D* = 0.076; *P* = 2 × 10^−6^). High-retention α copies retain >25% of ancestral CLG content relative to amphioxus (mean 38.9%; s.d. 4.8% across gar, chicken, and frog) while paralogous low-retention β copies retain <25% (mean 15.1%; s.d. 5.3%). The mean difference between the retention fractions of α–β pairs is significantly higher than a null model in which retention is uniformly random across pairs of chromosomes (*P* = 1.4 × 10^−5^; Supplementary Note 6). Notably, the assignment of vertebrate chromosome segments to their respective α and β columns in Fig. 3 based on high and low retention is consistent with the patterns of CLG fusion. Across orthologous segments, retention fractions are highly correlated between the spotted gar, chicken and frog (pairwise Pearson correlations: 94–96%), consistent with most gene losses occurring in the immediate aftermath of the α–β duplication before the divergence of bony vertebrates. In their analysis of vertebrate genome duplication, Smith et al.^15^ often only detected the α signal (Supplementary Note 6), accounting for the predominance of two rather than four paralogous copies in their study.

### The auto- then allotetraploidization model of vertebrate genome evolution

Our auto- then allotetraploidization model (Fig. 5) differs from previous proposals in both the timing and modes of genomic duplication during vertebrate evolution^3,11^. In our scenario, an initial 1R doubling occurred before the divergence of the lamprey and jawed vertebrate lineages via autotetraploidization, and was followed by a second duplication (here, called 2Rjv) in the jawed vertebrate lineage (that is, after the lamprey lineage had diverged) via allotetraploidization. Since Putnam et al.^10^ and Venkatesh et al.^46,47^ previously found extensive syntenic conservation between the genomes of the elephant shark and some bony vertebrates, we infer that the palaeo-allotetraploidy described here in bony vertebrates had already occurred before the last common gnathostome ancestor. A strong prediction of our model is thus that, when fully characterized, cartilaginous fish chromosomes will show the patterns of CLG fusion described in Fig. 3. Previous scenarios have suggested two rounds of allotetraploidization^3^ or two rounds of autotetraploidization^11^. Several studies based on gene trees suggested that two duplications preceded the lamprey–jawed vertebrate split^12,14^, although an earlier analysis could not resolve the position of the first duplication (1R) relative to this split^10^.

The 1R event shared by all vertebrates is analogous to the autopolyploidizations described in salmonids, cyprinids (carps and their relatives) and sturgeons (reviewed in refs. ^48,49^). One of the ensuing autotetraploid lineages gave rise to lampreys, which lack CLG fusions that are found across bony vertebrates (Extended Data Fig. 3). A second autotetraploid lineage, leading to the jawed vertebrates, experienced the series of chromosomal fusions described in Fig. 3. These rearrangements were probably associated with a period of genetic diploidization (that is, the transition from tetrasomic to disomic inheritance (the formation of consistent bivalents between specific homologous pairs))^38^. Two descendants of this second lineage later hybridized in an allotetraploidization event (2Rjv) to give the jawed vertebrate ancestor.

Although many vertebrate gene families do not follow a doubly bifurcating pattern, as expected in a simple 2R scenario^4,16^, Furlong and Holland^11^ have elegantly argued that this phenomenon could be explained by extensive homeologous recombination during two closely spaced autotetraploidies. This argument generalizes to our auto- then allotetraploidy model as long as the diploidization period following 1R extended into the α and β progenitors before 2Rjv. Such a long period of residual tetrasomy after 1R is plausible; in salmonid fish, polysomic inheritance (that is, ongoing homeologous recombination) has persisted for tens of millions of years^43,49^. Detailed analysis of Hox-bearing chromosomes by Lynch and Wagner^50^ suggests that they experienced two chromosomal crossovers early in vertebrate evolution. Interestingly, the Hox gene clusters are found on CLGB, with HoxA and HoxD found on α segments and HoxB and C on β segments. However, unlike most other CLGs, we cannot uniquely pair specific α and β copies based on chromosomal linkage in the absence of a pattern of paired fusion involving CLGB. This suggests that the ancestral Hox-bearing chromosomes (as well as CLGA, G and H) could have experiences a prolonged period of homeologous interaction and exchange, consistent with Lynch and Wagner’s finding.

Although here we have described 17 CLGs by comparing the chromosomes of amphioxus and vertebrates, confirming ref. ^10^, several previous studies based only on comparisons among vertebrate genomes have variously inferred the existence of 10–13 ancestral vertebrate chromosomes^13,15,18–20^. Comparisons among vertebrate genomes without reference to an outgroup such as amphioxus are likely to miss the fusions that we have documented here, since anciently fused regions appear as single conserved syntenic blocks in comparisons among jawed vertebrates. Neglect of the complex history of fusions and mixing after duplication, and the reduced power to detect significant conserved synteny among paralogous regions with extensive gene loss (that is, the β segments of Fig. 3), appear to account for the discrepancies between previously published characterizations of ancestral vertebrate proto-chromosomes based on comparisons among or within vertebrates and our analyses (see also Supplementary Note 6 for several specific case studies). It is also important to consider that different portions of a vertebrate chromosome can have different ancestry. Specifically when assessed in 50-gene windows rather than at the whole-chromosome scale, we find statistically significant CLG–chicken and CLG–lamprey associations that were not found in earlier whole-chromosome comparisons of the chicken and sea lamprey (Extended Data Figs. 6 and 7 and Supplementary Note 6). Remarkably, in contrast with refs. ^13,15^, we find that lamprey chromosomes can be grouped into 17 clusters that correspond to our 17 CLGs (Extended Data Fig. 8**;** compare with Fig. 5b of ref. ^13^). Finally, the original proposal^10^ of 17 CLGs has found renewed support from analysis of the reconstructed ancestral amniote karyotype^14^. However, we note that this study used the non-chromosomal draft assembly of the amphioxus genome^10^ for its outgroup, and so made the same assumptions as ref. ^10^.

## Conclusion

Our analyses imply a novel scenario for vertebrate evolution and the events that shaped vertebrate genomes (Fig. 5). First, we show that 17 ancient CLGs^10^ are stable chromosomal units and that relicts of these units are readily detectable as either intact micro-chromosomes or large chromosomal segments in vertebrates, amphioxus, and even molluscs. Second, the jawed vertebrate lineage experienced two temporally and mechanistically distinct genome-wide duplications. The first—an autotetraploidization (1R)—preceded the divergence of lamprey and jawed vertebrate lineages^51^ ~490 million years ago (Ma). On the jawed vertebrate stem lineage, 1R was followed by a series of chromosomal fusions that preceded a second genome-wide duplication. On the lamprey lineage, 1R was followed by fewer and largely distinct fusions. Other studies^52,53^ have suggested that additional large-scale duplication events occurred in the lamprey lineage (as indicated by the node with a question mark in Fig. 5a), consistent with the one-to-many conserved synteny that we observe between amphioxus and lamprey (Extended Data Fig. 3), and the many-to-many relationship found between lamprey and chicken chromosomes^13,15^. Ongoing diploidization in both lineages after 1R, including gene loss^38^ and homeologous recombination^11^, is consistent with the complex orthology relationships observed between lamprey and gnathostome genes (for example, refs. ^52,54^). Disentangling these additional duplications specific to either the lamprey or cyclostome lineages will require further study. Third, the observation of asymmetrical gene retention implies that the second whole-genome duplication in the jawed vertebrate lineage (2Rjv) was an allotetraploidization (whole-genome duplication after interspecific hybridization). This second duplication preceded^10^ the divergence of bony and cartilaginous fish (~438–465 Ma)^55,56^. Our bounds on the timing of 1R and 2Rjv suggest that the extensive Ordovician diversification^51^ of early jawless and armoured fish (443–485 Ma) probably occurred during the period of diploidization after 1R, which was marked by chromosomal fusions and rearrangements that allowed new regulatory linkages to be explored. Hybridization of two related 1R descendants accompanied by genome duplication then established the lineage that subsequently gave rise to all living jawed vertebrates.

## Methods

### Chromosome-scale genome assembly and annotation

To produce a chromosome-scale assembly of amphioxus, we (1) reassembled existing shotgun data and then (2) ordered and oriented the resulting assembly with in vitro and in vivo chromatin conformation capture data.

We used ARACHNE^57^ to assemble the approximately tenfold redundant Sanger whole-genome shotgun sequence that was previously generated^10^ from a single diploid Florida lancelet (*B. floridae*). As in ref. ^10^, the two highly divergent haplotypes were assembled apart. For the present assembly, we resolved these diploid redundancies with HaploMerger2 (ref. ^58^) to produce a single reference haplotype for further analysis (Supplementary Note 1). In the present assembly, the total assembled contig length improved from 480–489 megabases and the L50 contig length doubled from 25.6–52 kb.

To achieve a chromosome-scale assembly of amphioxus, we obtained long-range linkages using in vitro (Chicago library^24,25^) and in vivo (Hi-C) chromatin conformation capture libraries, as described in ref. ^25^ and Supplementary Note 2. We used the HiRise pipeline (Dovetail Genomics) to scaffold the haplomerged amphioxus assembly with both types of chromatin conformation capture data (Supplementary Note 3). The resulting assembly of 19 chromosomal scaffolds (accounting for 94.5% of the assembled sequence) was validated by constructing a genetic map from light shotgun sequencing of 96 progeny from an F1 cross (Supplementary Note 4).

The protein-coding genes of amphioxus were annotated using the EVM pipeline^59^, incorporating recent transcriptome data^60^ for *B. floridae* (Supplementary Note 5). The process yielded 28,192 protein-coding loci containing 5,108 distinct Pfam domains—an increase of 6.5% relative to the 4,797 Pfam domains in the annotation of the earlier sub-chromosomal assembly^10^.

### Mutual best-hit orthology

To develop sets of high-confidence orthologous genes between amphioxus and selected vertebrate genomes, we performed mutual (that is, reciprocal) best BLASTp searches (Supplementary Note 6). Mutual best hits provides a high-confidence set of orthologues with minimal additional bioinformatics processing, and was used to define CLGs and identify syntenically orthologous units between amphioxus and vertebrate genomes.

### Definition of 17 CLGs

We partitioned the amphioxus chromosomes into 17 CLGs using the data from Fig. 1 and Extended Data Fig. 2, as follows. First, we identified boundaries along the amphioxus chromosomes between blocks of distinct vertebrate conserved synteny. Consider only genes *i* in amphioxus with mutual best hits in the comparator species, and define **x**_*a*_(*i*) as the synteny indicator vector of gene *i* that takes the value of 1 if the gene has its orthologue in chromosome *a* of the comparator, and 0 otherwise. To identify boundaries at which the conserved synteny changes, we computed the left and right windowed averages of the synteny indicator vector:

$${\mathbf{X}}_a^{\rm{L}}\left( i \right) = \frac{1}{W}\mathop {\sum}\nolimits_{j = i - W + 1,i} {{\mathbf{x}}_a\left( j \right)} \,{\rm{and}}\,{\mathbf{X}}_a^{\rm{R}}\left( i \right) = \frac{1}{W}\mathop {\sum}\nolimits_{j = i,i + W - 1} {{\mathbf{x}}_a\left( j \right)}$$

The squared Euclidean norm of the difference $D\left( {i,i + 1} \right) = {\mathbf{X}}_a^{\mathrm{R}}\left( {i + 1} \right) + {\mathbf{X}}_a^{\mathrm{L}}\left( i \right)$ then measures the discontinuity of conserved synteny between genes *i* and *i* + 1. Using as comparators the vertebrates chicken, gar or frog, or the invertebrate scallop, and the window size *W* = 25, *D* shows spikes at discontinuous boundaries.

We identified local peaks in *D* as intra-chromosomal boundaries in amphioxus between distinct patterns of conserved synteny. Consistent with the patterns seen in Fig. 1 and Extended Data Figs. 2 and 3, no boundaries were detected for most amphioxus chromosomes, but we identified four such synteny breakpoints in BFL2 and one boundary each in BFL3 and BFL4. These boundaries are consistent among vertebrates and scallops and consensus positions are indicated by vertical dashed lines in Fig. 1 and Extended Data Figs. 2–4. In several cases, the synteny indicator vectors of the chromosomal segments defined by the above procedure were closely aligned with each other. In these cases, the amphioxus segments were combined into a single syntenic unit (see further discussion in Supplementary Note 6). The resulting 17 CLGs (defined by the amphioxus genes contained in the corresponding segments) agree with the 17 putative ancestral linkage groups defined by clustering megabase-scale scaffolds (Supplementary Table 7) based on statistically significant patterns of conserved synteny with humans^10^.

### Gene families

To allow for analysis of (unlinked) gene duplication in vertebrates relative to the chordate ancestor, we also constructed families of orthologous chordate genes through sequence-based clustering analyses, as described in ref. ^10^. For the purposes of assessing the retention of gene duplicates after whole-genome duplication (see below), we counted only one gene family member per chromosome. With this counting, linked (for example, tandem) gene duplications produced by local processes do not lead to increased retention values, but unlinked duplications plausibly created through chromosome or genome-scale events are counted.

### Distribution of CLG ancestry across vertebrate chromosomes

Visualization of the local CLG ancestry across vertebrate chromosomes (Fig. 2) was based on orthologous chordate gene families. The height of each coloured bar in Fig. 2 was determined by the fraction of genes with the corresponding CLG ancestry in a window of at least 20 genes. Since gene density varies across vertebrate chromosomes, windows can have different physical sizes on Fig. 2, which shows base-pair position along chromosomes. To subdivide chromosomes according to their CLG ancestry, we searched through each chromosome for the largest peak in D, as defined above. This search was iterated with the condition that additional breakpoints were at least 20 genes away from a previously determined breakpoint. This process produces a partitioning of each chromosome into windows of at least 20 genes with relatively homogeneous CLG ancestry. Note that multiple CLGs can contribute to the same vertebrate chromosomal region, since blocks of CLG ancestry can overlap due to fusion and subsequent mixing, as described in the main text.

### Significance testing of syntenic associations between amphioxus and vertebrate genomes

We tested for significant associations between amphioxus and vertebrate genomes using a variation of the method described in ref. ^10^ and later applied by Smith and Keinath^13^ in their comparison of lamprey linkage groups with jawed vertebrate chromosomes. The null hypothesis was that orthologous genes are randomly distributed across the two genomes, with a Bonferroni correction for the total number of pairwise tests. While Smith and Keinath^13^ used gene families defined by collecting high-scoring hits among lamprey and selected vertebrates, we used the 6,843 mutual best hits between amphioxus and each of the four bony vertebrates: spotted gar, chicken, frog and human. Mutual best hits provide a conservative set of orthologues. For mutual best-hit orthologues, the equivalent null distribution for the distribution of orthologues shared between chromosomes (or between windows within chromosomes) is hypergeometric (Supplementary Note 6). The significance of associations between CLGs and vertebrate chromosomes, including a multiple test correction for the number of associations tested, is shown in Extended Data Fig. 6.

Based on Figs. 1 and 2 and Extended Data Fig. 3, we noted that, especially for vertebrate macro-chromosomes, orthologues from a given amphioxus chromosome or CLG are not uniformly distributed along the sequence, but rather appear to be concentrated in sub-chromosomal windows. To test whether sub-chromosomal windows show significant enrichments relative to the null model, we applied the same hypergeometric test to sliding windows of 50 genes (Supplementary Note 6) and accounted for the increased multiple testing with a Bonferroni correction based on the number of window tests. The resulting *P* values are shown in Extended Data Fig. 7. We note that this is a more sensitive test than the chromosome-scale test of Smith and Keinath, since associations that are not significant at the chromosome scale can be significant when tested with 50-gene windows. In Fig. 3, asterisks represent significant associations (*P* < 0.01 after Bonferroni multiple test correction) between CLGs and 50-gene windows; whereas plus signs indicate significance using 50-gene windows (*P* < 0.05), 100-gene windows (*P* < 0.01) and/or at the whole chromosome level (*P* < 0.05). Rows of Fig. 3 are also strongly supported by paralogy within vertebrate genomes.

### Definition of retention fraction

After genome duplications, unlinked duplicates may be lost. The retention fraction for a CLG on a vertebrate chromosome or chromosome segment is defined as the ratio of the number of CLG orthologues (that is, gene family members) found on that chromosome to the number of CLG-defining genes in amphioxus. Since a gene can be retained in multiple unlinked copies, the total retention of a CLG across all chromosomes of a vertebrate species can exceed one. As noted above, gene families are defined such that linked duplicates (arising by tandem duplication) are not counted towards the retention fraction; only unlinked duplicates are relevant for analysis of whole-genome duplications except in special circumstances of translocations that combine paralogous chromosomes from the same CLG on the same vertebrate chromosome. These translocations are identified by parsimony and comparison among vertebrate genomes, as described in the main text.

### Significance testing of asymmetry between high and low retention

To assess the significance of the difference between paired high- and low-retention classes, we compared the high and low means across the spotted gar and chicken against a null model in which retention rates in cells of Fig. 3 were chosen at random (that is, with only one retention class). To capture the broad distribution of values, we used a uniform distribution ranging from 0–0.532 (twice the overall retention mean). We chose pairs from this distribution and assigned the larger value as α and the smaller value as β. The difference between group means was normally distributed (confirmed by one million bootstrap simulations), and the observed group mean difference (high − low) was 7.16 standard deviations from the null model mean (Supplementary Note 7). To provide a conservative estimate of significance, we excluded pairs for which no β segment was found in Fig. 3. These pairs were excluded since coding these missing β segments as having a retention rate of zero only increased the high–low group difference. Similarly, using a normal distribution of retention rates (with mean and variance computed from observations) produced a null distribution of high–low means that was even farther from observed differences, so our use of the uniform distribution was conservative.

### Reporting Summary

Further information on research design is available in the Nature Research Reporting Summary linked to this article.

## Supplementary information

Supplementary Information Supplementary notes 1–7.

Reporting Summary

Supplementary Tables Supplementary Tables 1–7.

## Data Availability

The raw data and genome assembly are available from the National Center for Biotechnology Information BioProject under the accession code PRJNA412957. Processed data are available from https://bitbucket.org/viemet/public.

## Extended data

**Extended data** is available for this paper at 10.1038/s41559-020-1156-z.

## Code availability

The custom code used in this study is available from https://bitbucket.org/viemet/public.
