# Supplementary material for: Deeply conserved synteny resolves early events in vertebrate evolution
Source: Nat Ecol Evol. 2020 Apr 20;4(6):820–30. doi: 10.1038/s41559-020-1156-z (PMC7269912; doi:10.1038/s41559-020-1156-z)
Supplement: Supplementary file 1 — Supplementary notes 1–7. [file 41559_2020_1156_MOESM1_ESM.pdf]

nature ecology & evolution
SUPPLEMENTARY INFORMATION
https://doi.org/10.1038/s41559-020-1156-z
In the format provided by the authors and unedited.

Deeply conserved synteny resolves early events in vertebrate evolution

Oleg Simakov[1,2,13] ✉, Ferdinand Marlétaz[1,11,13], Jia-Xing Yue[3,12], Brendan O'Connell[4], Jerry Jenkins[5], Alexander Brandt[6], Robert Calef[7], Che-Huang Tung[8], Tzu-Kai Huang[8], Jeremy Schmutz[5], Nori Satoh[9], Jr-Kai Yu[8], Nicholas H. Putnam[7], Richard E. Green[4] and Daniel S. Rokhsar[1,6,10] ✉

[1]Molecular Genetics Unit, Okinawa Institute of Science and Technology Graduate University, Okinawa, Japan. [2]Department of Neuroscience and Developmental Biology, University of Vienna, Vienna, Austria. [3]Université Côte d'Azur, CNRS, INSERM, IRCAN, Nice, France. [4]Department of Biomolecular Engineering, University of California, Santa Cruz, Santa Cruz, CA, USA. [5]HudsonAlpha Institute for Biotechnology, Huntsville, AL, USA. [6]Department of Molecular and Cell Biology, University of California, Berkeley, Berkeley, CA, USA. [7]Dovetail Genomics, Scotts Valley, CA, USA. [8]Institute of Cellular and Organismic Biology, Academia Sinica, Taipei, Taiwan. [9]Marine Genomics Unit, Okinawa Institute of Science and Technology Graduate University, Okinawa, Japan. [10]Chan Zuckerberg Biohub, San Francisco, CA, USA. [11]Present address: Centre for Life's Origins and Evolution, Department of Genetics, Evolution and Environment, University College London, London, UK. [12]Present address: State Key Laboratory of Oncology in South China, Collaborative Innovation Center for Cancer Medicine, Sun Yat-sen University Cancer Center, Guangzhou, China. [13]These authors contributed equally: Oleg Simakov, Ferdinand Marlétaz.
✉e-mail: oleg.simakov@univie.ac.at; dsrokhsar@gmail.com

Supplementary Notes

Note 1. Reassembly of Sanger shotgun sequences

1A. Reassembly of whole genome shotgun data

We used a modified version of Arachne v20071016 (Jaffe et al. 2003) to reassemble the previously reported whole genome shotgun datasets for *Branchiostoma floridae* (the Florida lancelet), with genomic DNA obtained from a single outbred individual (Putnam et al. 2008). These data include shotgun sequence from three ~3 kb insert libraries (4.84x), six ~6-8 kb insert libraries (4.72x), five fosmid libraries (0.72x), and two BAC libraries for a total of 10.3x coverage (across both haplotypes). The sequencing libraries are summarized in more detail in Supplementary Table 1. Assembly parameters were: correct1_passes=0 maxcliq1=160 BINGE_AND_PURGE=True. This produced a raw assembly consisting of 3,637 scaffolds (42,102 contigs) totaling 891.5 Mb of sequence that largely splits the two divergent haplotypes present in this shotgun sequence. The scaffold N50 length is 1.7 Mb, with 905 scaffolds longer than 100 kb accounting for 865.4 Mb (97.1% of total assembly). The raw assembly statistics are given in Supplementary Table 2.

1B. Haplotype reconciliation

Due to the extreme heterozygosity of diploid amphioxus (Putnam *et al.* 2008), Arachne splits most of the assembly into two haplotypes. In order to produce a chromosome-scale reference genome, we used HaploMerger2 (Release 20151124) (Huang et al. 2012; Huang et al. 2014) to separate the Arachne scaffolds into a set of reference and alternate haplotypes, as follows.

First, repetitive regions of the assembly were identified and masked with RepeatMasker (version open-4.0.5) (http://www.repeatmasker.org) (options: -lib bf_repeats.fasta -s -gff -xsmall). Repetitive regions were soft masked (*i.e.*, repetitive regions were marked using lowercase letters). NCBI rmblast (v2.2.27+) was used as the search engine for RepeatMasker. A previously curated *B. floridae* repeats library (Putnam *et al.* 2008; retrieved from http://genome.jgi.doe.gov/Brafl1/Brafl1.home.html) was used as a custom repeat library for RepeatMasker. According to the masking result, repetitive sequences constituted 23.88 % of the Arachne diploid assembly.

The masked Arachne diploid assembly was then processed with HaploMerger2 (Release 20151124) (Huang et al. 2012; Huang et al. 2017) to collapse the two haploid genomes. Briefly, HaploMerger2 examines the allelic relationship of scaffolds from the diploid

assembly by all-against-all whole genome alignment and subsequently reconstructs the reference and alternative haploid assembly based on reciprocal best scaffold alignments. During this process, mis-joins in the original diploid assembly and tandem mis-assemblies in the resulting haploid assemblies are also detected and rectified.

We used modules A-D of HaploMerger2 in our analysis. We used the default score matrix shipped with HaploMerger2, since this matrix was originally generated based on the *B. floridae* genome. We first ran module A (hm.batchA1-hm.batchA3) for three rounds to identify allelic relationship of the input scaffolds and remove possible mis-joins in the input diploid assembly. We removed both large-scale (>50kb) and medium-scale (30-50kb) mis-joins by jointly adjusting filtering thresholds for each round (aliFilter=5000000 & overhangFilter=50000 for the first round; aliFilter=4000000 & overhangFilter=40000 for the second round; and aliFilter=3000000 & overhangFilter=30000 for the third round). The output of the previous round was used as the input for the next round.

Next, we ran module B (hm.batchB1-hm.batchB5) to reconstruct haploid assemblies based on the final output of module A. This step resulted in two haploid assemblies to represent the reference and the alternative copy of the haploid genome sequences. These two haploid assemblies were fed into module C (hm.batchC1-hm.batchC2) for three rounds of scaffolding by referring to the original Sanger paired-end reads. Internally, module C called SSPACE standard (v3.0) (Boetzer et al. 2011) to perform scaffolding. A minimum of 5 read pairs are required for linking two contigs. The scaffolded haploid assemblies (both the reference and the alternative one) were further processed by module D (hm.batchD1-hm.batchD3) for three rounds with different filtering settings (filterAli=4000 & minLen=5000 for the first round; filterAli=2400 & minLen=3000 for the second round; and filterAli=1000 & minLen=1500 for the third round) to remove tandem assembly errors. Again, the output of the previous round was used as the input for the next round during this process. The summary statistics of the original diploid assembly generated by Arachne as well as the two final haploid assemblies coming out of the haplomerging process are provided in the Supplementary Table 3 below. After haplotype reconciliation, the scaffold N50 length was 2.8 Mbp.

Note 2. Chromatin conformation libraries and sequencing

2A. *In vitro* chromatin conformation capture

In vitro chromatin conformation capture libraries (“Chicago”) were prepared at Dovetail Genomics (Santa Cruz, USA) from high molecular weight DNA as described in (Putnam *et al.* 2016).

2B. “HiC” chromatin conformation capture libraries

Sample preparation, lysis, and immobilization. One whole *B. floridae* animal was dissected to remove the gut. The remainder of the tissue was crosslinked in 1% formaldehyde for 15 minutes at room temperature in 100 µl volume. The reaction was quenched with 2.8 µl 2.5M Glycine, and centrifuged to remove the excess formaldehyde. The animal was re-suspended in 550 µl lysis buffer (10mM HEPES pH-8.0, 10mM NaCl, 0.2% IGEPAL CA-630, and 1X Protease inhibitors solution (Roche)) and 250 µL 0.5mm Silica beads and vortexed at high speed for 5 minutes. The lysate was removed, pelleted by centrifugation at 2500rcf, and washed twice with 50 mM Tris.HCl pH=8.0, 50mM NaCl, 1mM EDTA). The sample was resuspended in 250 µl of the same buffer, before adding 95 µl 1% SDS as previously described in (Lieberman-Aiden et al. 2009). Chromatin was immobilized on SPRI beads at a SPRI-lysate ratio of 2:1 (Ma et al 2014), then washed with 10mM Tris, 50mM NaCl to remove non-histone-associated DNA.

*Restriction digestion***.** The bead/chromatin mixture was re-suspended in 49.5 µL 1X NEBuffer 2, and digested with 5 units of DpnII enzyme for one hour at 37°C in a thermal-mixer. After digesting, the beads were concentrated and washed twice with wash buffer.

End-labeling. The sample was resuspended in a 50 µL reaction containing dA-dT-and dGTP, biotinylated dCTP, and Klenow. End-labeling was performed at 25°C for 30 minutes, then the sample was washed twice with wash buffer.

Chromatin proximity ligation. The sample was ligated overnight in a 250 µL reaction containing 1X NEB T4 ligase buffer, 0.1mg/ml BSA, 0.25% Triton X-100, and 50 units T4 DNA ligase. After ligation, 2.5 µL 10 mM dNTPs and 5.5 units T4 DNA polymerase were added to remove un-ligated biotin-dCTP.

Crosslink reversal. After concentrating the sample and removing the ligation buffer, the crosslinks were reversed and the sample was de-proteinated in 50 µL cross-link reversal buffer (50mM Tris pH=8.0, 1% SDS, 0.25 mM $CaCl_2$, and 0.5 mg/mL Proteinase K). The sample was incubated at 55°C to digest the histones and other chromatin-associated

proteins, then the temperature was increased to 68°C to reverse the crosslinks. After cross-link reversal, the sample was separated from the beads and purified on fresh SPRI beads at a ratio of 2:1, following (Putnam et al. 2016). DNA recovery was quantified by Qubit fluorometer.

Library preparation. The sample was split into two replicates before library preparation. Illumina sequencing libraries were made using the NEB Ultra library preparation kit according to manufacturer's instructions, with one exception: prior to indexing PCR, the sample was enriched by pulldown on 30 µL Invitrogen C1 Streptavidin beads, then washed to remove non-biotinylated DNA fragments.

Note 3. Chromosome-scale assembly

The haplopmerged Sanger assembly, shotgun reads, Chicago library reads, and Dovetail HiC library reads were used as input data for HiRise, a software pipeline designed specifically for using proximity ligation data to scaffold genome assemblies (Putnam et al. 2016). An iterative analysis was conducted. First, Shotgun and Chicago library sequences were aligned to the draft input assembly using a modified SNAP read mapper (http://snap.cs.berkeley.edu). The separations of Chicago read pairs mapped within draft scaffolds were analyzed by HiRise to produce a likelihood model for genomic distance between read pairs, and the model was used to identify and break putative misjoins, to score prospective joins, and make joins above a threshold. After aligning and scaffolding Chicago data, Dovetail HiC library sequences were aligned and scaffolded following the same method. After scaffolding, shotgun sequences were used to close gaps between contigs. Nineteen assembled scaffolds are longer than 17 Mbp, and represent chromosomes, as summarized in Supplementary Table 4. The remaining scaffolds are all shorter than 1 Mbp and represent short sequences not assigned to a chromosome in our HiC assembly. The chromosome sequences account for 94.2% of the total assembly. This is consistent with 94.5% of annotated protein-coding genes (see Note 5) being assigned to chromosomes. According to BUSCO analysis (Simão et al. 2015) 95.8% of 978 representative single copy bilaterian genes are present in the assembly.

Note 4: Construction of amphioxus genetic map

To construct a genetic map for *B. floridae*, we crossed two unrelated parents and raised 96 of their F1 progeny until young adult stage. The parents of the cross were selected from a laboratory colony of amphioxus adults that were raised from embryos at the Institute of Cellular and Organismic Biology, Academia Sinica, Taiwan. This colony was derived from ~100 wild caught amphioxus adults from Tampa Bay, Florida, provided by Linda Holland and Nicholas Holland at the Scripps Institution of Oceanography, University of California at San Diego, and Daniel Meulemans Medeiros at the University of Colorado, Boulder.

DNA from the 96 progeny samples was prepared using the Maxwell Tissue DNA purification kit on a Maxwell Instrument (Promega). Illumina libraries were prepared and sequenced on four HiSeq4000 lanes in 2x150 mode, yielding an average 12M paired-end reads per individuals (nominal ~7x coverage). DNA from both parents of the cross was extracted using Phenol-Chloroform protocol and sequenced at nominal 70x depth on a HiSeq2500 instrument with rapid run mode enable 2x250 paired-end reads.

Parents and progeny were genotyped by whole genome shotgun sequencing. All reads were aligned to the chromosome-scale reference *B. floridae* assembly using BWA-MEM (Li and Durbin 2010), alignments were merged, and sorted, and duplicates were marked using novosort (Novocraft). Per site allele depths were extracted from the population from a VCF file of biallelic single nucleotide polymorphisms (SNPs) generated by FreeBayes v1.1.0 with a minimal alternate count of 3 reads (-C) and a minimal alternate fraction of 0.2 and filtered on quality (>20) (Garrison and Marth 2012). Genotyped sites in the male and female parents had median depth of 66x and 67x, respectively. Progeny were sequenced to an average depth of 6.9x base coverage, which after mapping and filtering yields a 3x depth for SNPs.

We constructed separate male and female meiotic linkage maps using the pseudo-testcross method (Grattapaglia and Sederoff 1994). For the male map, we used biallelic SNPs that were heterozygous in the father and homozygous in the mother, allowing transmission of paternal alleles to be tracked; conversely, for the female map we used biallelic SNPs that were heterozygous in the mother and homozygous in the father. Sites that were heterozygous in both parents were ignored. This filter resulted in 459,883 biallelic SNP markers segregating from the father, and 423,361 from the mother, for a total of 883,244 segregating markers.

At an average progeny depth of 3x, we could reliably positively detect heterozygous progeny (*i.e.*, progeny that inherited the minor allele in the cross from the heterozygous parent) but progeny with only a few observed major allele-bearing reads could be either

homozygotes, or heterozygotes for which the minor allele was simply not sampled. These "rough" genotypes are not themselves directly suitable for linkage map construction, but can be used to impute high confidence genotypes using linkage.

To robustly call genotypes, we used the fact that nearby sites (*e.g.*, within 1 Mbp) are strongly linked, and are therefore either strongly correlated (minor alleles "coupled" on the same haplotype in the heterozygous parent) or anti-correlated (minor alleles "in repulsion" found on opposite haplotypes in that parents). We developed a simple algorithm (Brandt *et al.*, in preparation) to impute genotypes at each site based on the correlations observed among the rough initial genotypes at sites within a 500 kbp window along the assembly. Briefly, consider the graph with each node representing a segregating site in the genomic window, and each edge representing the correlation between the rough genotypes at these sites. We only consider edges that connect sites at which the rough genotypes are either concordant (coupled) or discordant (in repulsion) in a net of four of the 96 progeny.

We constructed a minimum spanning tree of edges among nodes in the window, minimizing the sum of the negative absolute value of Pearson correlation coefficients along the spanning tree. The sign of the correlations along the tree allows us to determine the relative phase of each SNP inherited from the heterozygous parent. This analysis determines the two haplotypes of the heterozygous parent within each 500kb window. Progeny are assigned one of the two haplotypes if (1) the rough genotypes in the window agree with one of the two haplotypes at five or more sites, and (2) no more than 20% of the rough genotypes are discordant with that haplotype.

Markers for paternal meiotic map. Out of 970 non-overlapping full 500 kb genomic windows along the 19 chromosomes, 771 (79.5%) contained sufficiently many correlated paternal SNPs (*i.e.*, at least five, with no more than 20% discordance, see above). Of these, 702 could be called in more than 80% of progeny, and were used for paternal map construction. In addition, 309 windows were shorter than 500 kb (representing ends of contigs/chromosomes, and sub-500kb scaffolds). Of these windows, 128 (41.4%) had sufficiently many correlated paternal SNPs. Of these, 47 could be called in more than 80% of progeny.

Markers for maternal meiotic map: Out of 970 non-overlapping full 500 kb genomic windows along the 19 chromosomes, 780 (80.4%) contained sufficiently many correlated maternal SNPs. Of these, 713 could be called in more than 80% of progeny, and were used for maternal map construction. Of the 309 windows shorter than 500 kb, 124 (40.1%) contained sufficiently many correlated maternal SNPs. Of these, 40 could be called in more than 80% of progeny.

We constructed separate male and female linkage maps with the OneMap package (v2.0-3) in R (Margarido, Souza, and Garcia 2007), constructing each map using the “F1 cross” setting and providing the genotype calls for non-overlapping 500 kb windows as described above. We found 19 major linkage groups in both maps (log odds (LOD) threshold 5 for male map and 10 for female map). Additional markers not meeting these thresholds were then placed on the linkage groups using “forced” mode, with LOD placement threshold 3, using three seed markers for the male map and seven seed markers for the female map. The “touchdown” setting was used to place remaining markers with a LOD placement threshold of 2.

The 19 male and female linkage groups are in 1:1 correspondence with the 19 chromosome-scale scaffolds assembled using HiC chromatin linkages, confirming the accuracy of these chromosomes. The total length of the 19 linkage groups of the male and female maps were 756.8 and 950.8 cM, respectively. Although we genetically confirmed the chromosomal linkages of our HiC-based assembly, the ordering of markers was not perfectly concordant with their order along the assembly. In particular, the assembly of chromosome 19 appears to have an intrachromosomal rearrangement relative to the genetic map, suggesting need for further refinement. Since our major results depend on amphioxus linkage but are insensitive to the intra-chromosomal gene order, we used the HiC-based assembly of *B. floridae* for all comparative analyses.

Note 5. Annotation of protein-coding genes

We generated a new annotation of the protein-coding genes of amphioxus with the EVM (Haas et al. 2008) pipeline. Genbank ESTs, and a Trinity assembly of recently published amphioxus RNAseq data (Hu et al. 2017), were aligned to the genome using PASA (Haas et al. 2003). Putative coding regions within assembled transcripts were detected using trans-decoder (Haas et al. 2008).

To construct a training set for the Augustus gene prediction algorithm (Stanke et al. 2006) we collected complete open reading frames (ORFs) from assembled transcripts that showed (i) similarity against Swissprot (e-value < 1e-6) and (ii) at least 3 exons. A set of *de novo* gene predictions was obtained using the newly trained Augustus profile, and were subsequently validated and refined using EVM (Haas *et al*. 2008) incorporating the original PASA transcript alignments, as well as alignments of proteins from human and previous *B. floridae* annotation generated with exonerate (Slater and Birney 2005).

This process yielded 28,192 loci containing a total 5,108 distinct PFAM domains, in comparison to the 4,797 found in the original *B. floridae* annotation (Putnam et al. 2008). The annotation described above was performed on an intermediate assembly (2uHHq) that had the same underlying contig sequence, but with different scaffolding. Annotated genes were transferred to the final assembly using exonerate (Slater and Birney 2005).

Note 6. Characterization of deep synteny

6.1 Genome datasets used

Human; *Homo sapiens*: Ensembl 78
Frog; *Xenopus tropicalis* chromosomes: Xenbase v9
Chicken; *Gallus gallus*: Ensembl v5
Spotted gar; *Lepisosteus oculatus*: Ensembl LepOcu1 (Braasch et al. 2016)
Amphioxus; *Branchiostoma floridae*: this study
Acorn worm: *Saccoglossus kowalevskii:* Simakov et al. 2015
Owl limpet; *Lottia gigantea*: Simakov et al. 2013
Drosophila; *Drosophila melanogaster*: Ensembl 78
C. elegans; *Caenordhabditis elegans*: Ensembl 78
Sea lamprey; *Petromyzon marinus*; Smith et al. 2018
Scallop: *Patinopecten yessoensis*; Wang et al. 2017.
European amphioxus: *Branchiostoma lanceolatum*; Marletaz et al. 2018.

6.2 Orthologous gene families anchored by mutual best hits

We identified 6,843 groups of orthologous genes anchored by mutual best BLAST hits between amphioxus and each of four well-assembled chromosome-scale jawed vertebrates used in our analysis (chicken, spotted gar, frog, and human). These gene families were extended to include paralogs using the phylogenetically informed method described in Simakov et al. 2013. To avoid biasing our analyses due to linked paralogs (most of which are recent tandem duplications relative to the ancient chromosome-scale events of interest), we considered only a single paralog per chromosome in Figs. 1 and Extended Data 2. We used mutual best hits, without considering paralogs, for comparisons between amphioxus and invertebrate genomes (Extended Data 4) and lamprey (Extended Data 3).

6.3 Comparison with draft assembly of European amphioxus

Recently, Marletaz et al. (2018) have reported a sub-chromosome-scale assembly of the European amphioxus *B. lanceolatum* with N50 length 1.6 Mbp. Although the scale of this assembly is shorter than our pre-HiC assembly of *B. floridae* (N50 length 2.8 Mbp) we can use it to provide further corroboration of the local accuracy of our *B. floridae* assembly. Since the two *Branchiostoma* species diverged ~100 Mya, interpreting the *B. floridae*-*B. lanceolatum* comparison depends on the assumption that over such "short" time scales synteny is preserved despite local inversions and other rearrangements.

Considering the 6,843 *B. floridae* genes with clear vertebrate orthologs that are used in the analyses described below, we find that 65% of their *B. lanceolatum* orthologs are found on 123 scaffolds bearing at least 10 such genes. Of these scaffolds, 119 (96.7%) can be

uniquely assigned to a single *B. floridae* chromosome, allowing for only local rearrangements. The 4 discrepant scaffolds could correspond to *bona fide* rearrangements between the *Branchiostoma* species but are more likely to be assembly errors in *B. lanceolatum*. We note that our *B. floridae* assembly has validation from BAC- and fosmid-end mate pairs as well as both *in vitro* and *in vivo* HiC data; such confirmatory data was not available for the European amphioxus work. Therefore a small number of long-range errors in the draft *B. lanceolatum* assembly would not be unexpected.

6.4 Identification of 17 Chordate Linkage Groups

Based on visual examination of the dotplots (both MBH and clustering-based) between amphioxus and other species, we observed consistent breakpoints of conserved synteny on three amphioxus chromosomes (2, 3, and 4) compared with vertebrate species and with scallop. These breaks, and grouping the resulting segments according to shared synteny, results in 17 amphioxus-vertebrate shared linkage groups (CLGs). The positions of these boundaries were determined as follows.

First, we identified boundaries along the amphioxus chromosomes between blocks of distinct vertebrate conserved synteny using data shown in **Fig. 1** and **Extended Data 2**. Consider genes i in amphioxus with mutual best hits in a comparator species. We define $x_a(i)$ to be the 'synteny indicator vector' of gene i that takes the value of 1 if the gene has its ortholog in chromosome a of the comparator, and 0 otherwise. In order to identify boundaries at which the conserved synteny changes, we computed the averages of the synteny indicator vector over left and right windows of length W = 20 (up to and including site i):

$$X^{L}_{a}(i) = (1/W)\ \Sigma_{j=i-W+1,i}\ x_a(j) \text{ and } X^{R}_{a}(i) = (1/W)\ \Sigma_{j=i,i+W-1}\ x_a(j).$$

The squared Euclidean norm of the difference $D(i, i+1) = X^{R}_{a}(i+1) - X^{L}_{a}(i)$, which measures the discontinuity of the average synteny indicator vector x_a between genes i and $i+1$. $|D|^2$ shows sharp spikes at the discontinuities in synteny evident in **Fig. 1** and **Extended Data 2**, and allows a precise determination of these boundaries.

Consistent with the patterns seen in **Fig. 1** and **Extended Data 2** and **3**, no discontinuities were detected for most amphioxus chromosomes, but we identified four such synteny breakpoints in BFL2 and one boundary each in BFL3 and 4. These boundaries are consistent among vertebrates and scallop; consensus positions are indicated by vertical dashed lines in **Fig. 1** and **Extended Data 2-4.** When the amphioxus segments defined by this procedure had the same pattern of vertebrate synteny, their indicator vectors were closely aligned. In these cases the amphioxus segments were combined into a single CLG. **Supplementary Table 6** summarizes the boundaries of the CLG-defining segments.

We note that, despite having the same *chromosomal* pattern of conserved synteny across jawed vertebrates (Figs. 1 and Extended Data 2), CLGI and CLGQ are represented as distinct ancestral units. CLGI and CLGQ are considered distinct because they not only occur as distinct chromosomes of both outgroups amphioxus and scallop (Figure 1), but (1) they also exist as distinct units in lamprey (Extended Data 3 and 8), and (3) orthologs of these two amphioxus chromosomes are found in distinct regions on jawed vertebrate chromosomes (most easily seen in our Figs 1 and 2). This is in contrast to CLGB, which as noted in the main text is represented as a single ancestral unit despite being spread over three distinct chromosomes of amphioxus (BFL10, 16, and 18) and homologous chromosomes of scallop. CLGB is represented as a single ancestral unit because (1) the three amphioxus (and scallop) chromosomes have the same pattern of synteny in lamprey (Extended Data 3a) and (2) are found mixed over the same chromosomal regions in jawed vertebrate chromosomes (Figs. 1 and Extended Data 2).

In total, we infer 17 CLGs, consistent with the previous estimates in (Putnam et al. 2008) based on clustering of megabase-scale amphioxus scaffolds based on statistically significant patterns of conserved synteny with human. CLGs were assigned a letter A-Q in decreasing order of the number of chordate gene families they contain (based on amphioxus-vertebrate mutual best hits as defined above). These CLGs are in 1:1 correspondence with the 17 “putative ancestral linkage groups” (PALs) defined by clustering megabase-scale scaffolds based on statistically significant patterns of conserved synteny with human[10] (**Supp. Supplementary Table 7**).

6.5 Significance testing of blocks of conserved synteny

Previously, Smith and Keinath (2015) and *Smith et al.* (2018) argued that relatively few chromosomal comparisons between sea lamprey and bony vertebrates (*e.g.*, chicken (Smith and Keinath, 2015) and chicken and spotted gar (Smith *et al.*, 2018)) are significantly enriched for shared orthologs when compared with a null model, leading to their rejection of the "2R" hypothesis and development of a model in which jawed vertebrate chromosomes arose through a combination of individual chromosome-scale duplications preceding a single genome-wide event.

From our main Figs. 1 and 2, however, it is evident that, especially for macro-chromosomes, orthologs are typically enriched across only portions of these large vertebrate chromosomes. Significance tests based on interspecific comparisons of whole chromosomes to whole chromosomes are therefore likely to be under-powered, since enrichments confined to a portion of a large chromosome will be diluted when considered on a chromosome scale. (The statistical power of Smith-and-Keinath-style analyses may also reduced by the use of clustered lamprey chromosomes as the units of comparison, as the lamprey orthologs of jawed-vertebrate genes appear to be distributed over multiple

homeologous lamprey chromosomes due to additional genome duplications in the lamprey (or cyclostome) lineage.)

To test for segmental enrichments taking into account the apparent structure of vertebrate chromosomes relative to the chordate ancestor, we used sliding windows of m=50 (or 100) genes across vertebrate chromosomes. Importantly, the boundaries of the tested windows are chosen without regard to the boundaries visually seen in Figures 1 and 2, to avoid biasing the calculation. For this analysis we consider 6,843 mutual best hits shared between amphioxus, and chicken, spotted gar, human, and frog (Supp. Note 6.2), These gene families serve as an approximation to the gene content of the amphioxus-vertebrate ancestor. For this analysis we did not include paralogs. We note that the use of only mutual best hits may reduce the power of our calculation, but significance found using mutual-best-hits is an upper bound on significance calculated using more complex definitions of orthology. Comparison of testing between chromosomes and windows are shown in Extended Data 6 and 7 respectively.

Mutual best hits are unique in both amphioxus and the comparator vertebrate genome, so the relevant distribution of shared orthologs is given by the hypergeometric distribution. For any given window W_i of some predefined length, and a chordate linkage group L_i, the number of genes found within these two regions, relative to those found outside these regions can be computed using the one-tailed test for the hypergeometric distribution (which is conveniently isomorphic with Fisher's exact test). This is computed according to the following table:

	Inside *Li*	Outside *Li*
Inside *Wi*	*a*	*b*
Outside *Wi*	*c*	*d*

The p-values computed in this manner are scaled by a Bonferroni correction to account for the total number of windows tested.

Extended Data 7 shows the number of shared mutual best hits between each CLG and 50 gene windows of four bony vertebrates (chicken, spotted gar, frog, and human). The numbers of shared mutual best hits are shown as circles of proportional area, with significant windows (Bonferroni-corrected $p < 0.05$) shown in red. This analysis clearly shows that all CLGs have three or more significant windows of conserved synteny, contrary to Smith and Keinath's chromosome-chromosome comparisons (shown in Extended Data

6 for comparison). This analysis shows that Smith and Keinath's rejection of the 2R scenario based on the scarcity of evidence for three and higher paralogous blocks is flawed.

Since lamprey chromosomes are typically homogeneous in their CLG ancestry, it is instructive to show the same plot comparing CLGs to lamprey-chromosomes (Extended Data 8). It is clear from this figure that (1) the sea lamprey chromosomes form 17 natural groups according to the 17 CLGs, and (2) there are 6-8 lamprey chromosomes for each CLG, reflecting additional genome duplications in the sea lamprey lineage.

6.6 Distribution of CLG ancestry along vertebrate chromosomes

To display the regional CLG ancestry of bony vertebrate chromosomes, we represented the local fraction of CLG ancestry over non-overlapping windows of at least 20 genes by a stacked bar-chart (Fig 2). The boundaries of the windows were chosen using the synteny discontinuity indicator D(i, i+1) described above, now applied to each bony vertebrate chromosome with the 17 CLGs as comparators. Window boundaries were chosen as local maxima of D by an iterative process. The first boundary cuts the chromosome into two segments across genes (*i*, *i*+1) with maximal D. Each segment is then searched for an internal local maximum of D subject to the condition that it is at least 20 genes away from a previous boundary. This process is iterated until no further subdivision is possible.

6.7 Comparison of CLGs to previous studies

Our 17 chordate linkage groups are in 1:1 correspondence with the 17 putative ancestral linkage (PAL) groups obtained by Putnam et al. (Putnam et al. 2008) based on clustering of a non-chromosomal assembly of the amphioxus genome. In that analysis, megabase-scale amphioxus scaffolds were clustered based on the distribution of orthologous genes on human chromosomes. The 1:1 correspondence between Putnam et al. and the present work shows that scaffold-scaffold linkages predicted using this clustering approach are observed in the chromosomes of amphioxus, although we find that the PAL clusters of Putnam et al. do not have a 1:1 correspondence with chromosomes (see Fig. 1 and main text).

Nakatani et al. (Nakatani et al. 2007) predicted vertebrate linkage groups (VLGs) by analysis of intra-jawed-vertebrate conserved synteny. Similarly, Smith and Keinath, 2015 predicted ancestral vertebrate chromosomes (Anc elements) by comparing the chicken genome with a draft assembly of lamprey; many of these Anc elements were also supported by the later chromosome-scale lamprey genome assembly of Smith et al. Like our 17 CLGs (and Putnam et al’s earlier PALs) these VLG/Anc elements are attempts to infer chromosomal linkages on the vertebrate stem prior to any whole genome duplications.

Since our analyses find 17 CLGs, more than with the 10-13 linkage groups found by Nakatani et al. and Smith et al., our CLGs cannot be placed in complete 1:1 correspondence with these earlier predictions, which were made without reference to a chromosome-scale outgroup to the vertebrates. While some of our CLGs (and the earlier PALs of Putnam et al.) correspond directly to VLG or Anc elements, VLGs and Anc elements are often mixtures of our CLGs. Partial correspondence is provided in Supplementary Table 7.

6.8 Further discussion of two case studies and 2R vs 1R scenarios

It is instructive to consider in more detail two specific cases raised by an anonymous reviewer that highlight differences between our analysis and that of Smith et al. (2018). This discussion helps to understand two discrepancies: first, the difference between the 17 CLGs that we derive here using amphioxus and scallop as outgroups (first proposed by Putnam et al. 2008), vs. the 10-13 ancestral units claimed by previous studies (Nakatani et al. 2007; Smith and Keinath 2015; Smith et al. 2018), and second, the difference between our auto-then-allo-tetraploidy 2R scenario and Smith et al.'s model of a single whole genome duplication accompanied by smaller-scale duplications.

Case study #1: Vertebrate history of CLGE and CLGO. As an alternative scenario for our main Figure 4, the reviewer suggested that a fusion of CLGO and CLGE could have occurred prior to two rounds of whole genome duplication, and that such a scenario would also be consistent with conserved synteny blocks of Figure 4 of Smith et al. (2018) (see also Figure 2 of Smith and Keinath (2015)) because chicken chromosomes 1, 5, 12, and 26 share a significant number of orthologs in common. This scenario can be rejected.

First, careful consideration of Figure 4 of Smith et al. (2018) shows that *there is no single lamprey super-scaffold that shares significant conserved synteny with GGA1, 5, 26, and 12*. (Such a lamprey super-scaffold would appear as a horizontal row of dots in all four columns.) This observation is consistent with our Extended Data 8, which clearly shows that lamprey chromosomes are associated *either* with CLGE *or* with CLGO, *but never with both*. Thus any CLGE-CLGO fusion must have occurred *after* the divergence of lamprey from the jawed vertebrate lineage.

We also note that the special relationship between GGA12, 26 and 1 suggested by light red shaded box in Figure 4 of Smith et al. (2018) is somewhat misleading. Smith et al. use the trio of chicken chromosomes GGA12, 26, and 1 (light red shaded box) as evidence for a single round of whole genome duplication combined with chromosome-scale duplication (since in their analysis there are only three chromosomes involved in this "Anc" unit). GGA5 is not included in this red shaded box despite its significant conserved synteny (via one lamprey super-scaffold) with GGA1 and GGA26, but is instead grouped with GGA3 (light blue shaded box) in Smith et al (2018).

In contrast, from our analyses (see our Figures 1-3) we see that

(1) GGA1 and GGA26 comprise an "α-β" pair, and each of these chicken chromosomes contain a partially intermixed copy of *both* CLGE *and* CLGO.
(2) GGA12 has a significant contribution from CLGE *but not CLGO*. It is classified as an "α" segment based on gene retention relative to the amphioxus outgroup. The corresponding "β" segment is found on chromosomes LOC1 and XTR8 in spotted gar and Xenopus, respectively, but has been lost or is undetected by our analysis in chicken. Consistent with our model, this "β" segment is associated with CLGE only.
(3) GGA5 has a significant contribution from CLGO *but not CLGE*. It is classified as an "α" segment based on gene retention relative to the amphioxus outgroup. The corresponding "β" segment has been lost or is undetected by our analysis.

These three observations, coupled with the demonstration that lamprey super-scaffolds are either associated with CLGE or with CLGO but not both, supports out scenario shown in Figure 4: (1) an initial "1R" duplication of CLGE and CLGO when they were distinct units; (2) divergence of lamprey at this stage, resulting in multiple lamprey chromosomes that are either associated with CLGE or CLGO but not both; (3) fusion of one CLGE and one CLGO copy along the jawed vertebrate stem, with the other copies unfused; (4) a second "2Rjv" event that duplicated (a) the CLGE-CLGO fusion (to produce a portion of GGA1 and all of GGA26) as well as (b) the unfused copy of CLGE (to produce GGA12 and another segment lost or undetected in chicken but found in gar and frog). The unfused copy of CLGO from 1R is found in only a single copy (GGA5); the inference of a missing or lost second copy from 2Rjv is made based on the consistency of this scenario extended to the entire genome.

The presence of blocks of CLGE and CLGO ancestry on XTR4. As an aside related to the CLGE/CLGO discussion, we note that the frog chromosome XTR4 has contributions from *both* CLGE and CLGO, yet as noted in Figures 3 and 4 this is *not* considered evidence that this chromosome descended from a CLGE-CLGO fusion. The distinct pink (CLGO) and green (CLGE) syntenic blocks on XTR4 are on opposite ends of the chromosome (Figure 2), and are not juxtaposed/mixed as on *bona fide* E-O fusion chromosomes. Furthermore (as indicated in Figures 3 and 4) the CLGE and CLGO segments on XTR4 are orthologous to segments on different chromosomes of both chicken (Extended Data 5) and spotted gar (not shown). This supports the statement that their co-location on XTR4 represents a fusion in the *Xenopus* lineage after divergence from chicken, and that the ancestral state of bony vertebrates is for these particular copies of CLGE and CLGO to be unlinked.

We note that since *Xenopus* has only ten chromosomes (all macrochromosomes), many lineage-specific fusions are required to reach this small chromosome number starting from a much larger number of ancestral jawed vertebrate units (at least 30, even in the Smith et

al. scenario). So it is not surprising to find that such lineage-specific fusions have convergently brought together a given CLG pair independently of the stem fusions described in Figure 3. This is also consistent with the orthology of these two segments to different chromosomes in both chicken and gar as noted above.

Case study #2: Vertebrate history of CLGI and CLGQ. Another instructive case raised by the anonymous reviewer is that of CLGI and CLGQ, a pair of CLGs that are linked in all three of gar, chicken, and frog. Specifically, these appear together as IQ and FIQ on GGA4, 6, 13, and 22 as seen in Figure 3 and Fig. 2. The anonymous reviewer suggests that since there are lamprey super-scaffolds that have common orthologs with GGA4, 6, 13, and 22 (Fig. 4 of Smith et al. (2018)), a CLGI-Q fusion could have predated the divergence of jawed vertebrates and lamprey.

While it is true that there are lamprey super-scaffolds that have common orthologs with GGA4, 6, 13, and 22 (Fig. 4 of Smith et al. (2018)), and that these chicken chromosomes contain descendants of both CLGI and CLGQ genes (our Figure 3, and Figures 1 and 2), we note that *there are no lamprey super-scaffolds on which CLGI and CLGQ orthologs co-occur more than expected by chance*, as shown in our Extended Data 3b and 8. We conclude that *CLGI and CLGQ are unlinked in lamprey and amphioxus (and scallop) and therefore existed as separate units early in vertebrate evolution*. This is why they are regarded as distinct CLGs (see Supp. Note 6.4 above).

The logical flaw in Smith and Keinath (2015) and Smith et al. (2018) is that chicken is *not a proper outgroup to the vertebrates, nor a suitable surrogate, since it is both (doubly) duplicated, and its lineage has experienced fusions since the divergence from the lamprey lineage*. Chicken macro-chromosomes are shown by our analysis to be fusions of ancestral units (Fig. 2 and 3). Using a *bona fide* outgroups like amphioxus and scallop, however, reveals the patterns shown in Figure 3 showing the history of duplication, fusion, and duplication again.

We note that even in Smith et al. GGA4 and 13 show a closer relationship to each other, since there are multiple lamprey scaffolds with conserved synteny with GGA4 and 13 but not GGA6 and 22. This is because (in our scenario) GGA4 and 13 also share CLGF ancestry, and the lamprey scaffolds that show conserved synteny with them are CLGF descendants.

One round of genome duplication, or two? In their analysis comparing the lamprey and chicken genomes, Smith and Keinath (2015) and Smith et al. (2018), observe many *pairs* of paralogous chicken chromosomes (see, e.g., Figure 4 of Smith et al. (2018) and Figure 2 of Smith and Keinath (2015)). Smith et al. take this preponderance of pairs as evidence for just a single whole genome duplication during vertebrate evolution, and account for the

rarer trios and quadruples they find by appealing to additional smaller scale (chromosome or sub-chromosome) duplications.

As shown in Figure 3, however, according to our scenario jawed vertebrate genomes typically contain three or four copies of each CLG. Taken as a whole, our analysis is consistent with two temporally and mechanistically distinct whole genome duplications in the jawed vertebrate lineage (summarized in our Figure 5). According to this model we classify jawed vertebrate copies as "α" or "β" based on their gene retention after the jawed-vertebrate-specific allotetraploidy, 2Rjv. Since the 1R allotetraploidy preceded 2Rjv, there are two α-β sets for each CLG, denoted "1" and "2." Due to their more extensive gene losses, "β" segments contain fewer genes than "α" segments, and are therefore more difficult to detect; some may even have been lost in jawed vertebrate evolution. The ability to detect "β" segments using comparisons between chicken and lamprey is further degraded by the fact that lamprey chromosomes have also experienced independent gene losses due to additional lamprey-specific segmental or genome-wide duplications after 1R.

If there were two whole genome duplications in the jawed vertebrate lineage, why did Smith et al. find so many paralogous pairs of chicken chromosomes, and so few triples and quadruples? Consulting our Figure 3 we see that the paralogous pairs of chicken chromosomes highlighted by Smith et al. are all 1α-2α c : GGA14-18 (our CLGH); GGA3-5 (CLGA); GGA17-8 (CLGM); GGA15-19 (CLGG); and GGA21-9 (CLGP). In all of these cases we find one or two additional paralogous "β" segments in the chicken genome not detected by Smith et al. (Figure 3). We are able to identify these additional segments because (1) we use the unduplicated amphioxus as an outgroup, which is not subject to lamprey-specific gene losses, and (2) we take into account the fusions evident in Figure 2, recognizing that many chicken chromosomes are segmental composites of ancestral units (CLGs). The results that Smith et al. use to support their 1R-plus-additional-duplication model can therefore be readily explained in our 2R scenario.

6.9 Co-linearity analysis

Although our analyses are dependent only on conserved linkages between amphioxus and vertebrate genomes, we also analysed conservation of colinearity. To measure the retention of co-linearity (same order of orthologous genes in two or more species) among chordate genomes, we applied Spearman rank coefficient for windows of 20 orthologous genes and step size of 10 genes for all pair-wise species comparisons among vertebrates and amphioxus. We called a block of 20 genes co-linear if the correlation was higher than 0.75. Supplementary Table 5 represents the amount of co-linear regions as a proportion of all regions that could be assessed. As expected, we observe a decline in co-linearity level with the increasing phylogenetic distance. Highest co-linearity is observed between

human-chicken. Amphioxus shares less than 5% co-linear regions with (jawed) vertebrates.

Note 7: Asymmetric retention

We compute the retention rate of a vertebrate chromosome relative to each of its component chordate linkage groups (CLGs) as the number of gene families from that CLG that are represented on each vertebrate chromosome divided by the total number of gene families from that CLG. For these purposes, we allowed at most one orthologous gene per vertebrate chromosome (i.e., retention estimates do not include tandem or other linked duplicates). Only orthologous groups assignable to a CLG were considered, i.e., belonging to the 6,843 orthologous gene families described in Supp. Note 6.2. Retention rate generally match the patterns observed on the dotplot. We observed a “background” retention rate of <0.05 for CLG-vertebrate chromosome combinations for which the vertebrate chromosome was not derived from that CLGs. This number likely corresponds to the average orthology mis-assigment and/or translocation rate of genes among chromosomes.

Plotting the distributions of retention rates in frog, chicken, and gar across CLGs, we observe two peaks α (38.9% $\pm$ 4.8%) and β (15.1% $\pm$ 5.3%), see Figure 4b. Assigning jawed vertebrate chromosomal segments to these two peaks partitions jawed vertebrate genomes into α and β subgenomes. As diagrammed in Figure 5 these represent the chromosomal descendants of two progenitors that combined via allotetraploidy to form jawed vertebrates. Differential gene loss after allotetraploidy has been found in paleo-allotetraploid plants (see Garsmeur et al. 2014) and in the paleo-allotetraploid *Xenopus laevis* (Session et al. 2016).

Significance testing

We tested the hypothesis that paralogs are retained asymmetrically among sub-genomes against a null hypothesis in which paralogs are retained at random. Pairs of retention values were selected at random from a uniform distribution between 0 and 0.532 (to ensure that overall retention mean is the same). For each pair, the larger value was designated "α" and the smaller value "β." For CLGs for which no fusions have been documented (Figure 3), we cannot relate a specific "α" segment to a particular "β" counterpart. To simulate these, we take four retention values from the normal distribution, and assign the top two to "α" segments and the bottom two to "β" segments. In this way, we construct a simulated version of Figure 3 from an explicitly symmetrical model in which the difference between "α" and "β" do not arise from any inherent asymmetry in the retention process.

We used the difference between high retention ("α") and low retention ("β") rates averaged over all pairs of chromosomal descendants of CLGs as a test statistic. To determine the distribution of this test statistic under the null model, we computed it for one million simulations. This empirical bootstrap distribution is normally distributed and

the high-low test statistic is 7.16 standard deviations from the mean. We therefore reject the null hypothesis with $p < 10^{-6}$, and conclude that the retention rates are asymmetrically distributed, supporting an allotetraploid model for the second duplication in the 2R scenario.

References

Boetzer, Marten, Christiaan V Henkel, Hans J Jansen, Derek Butler, and Walter Pirovano. 2011. "Scaffolding Pre-Assembled Contigs Using SSPACE." *Bioinformatics* 27 (4): 578–79.

Edgar, R C. 2004. "MUSCLE: A Multiple Sequence Alignment Method with Reduced Time and Space Complexity." *BMC Bioinformatics* 5. Department of Plant and Microbial Biology, 461 Koshland Hall, University of California, Berkeley, CA 94720-3102, USA. bob@drive5.com: 113. doi:10.1186/1471-2105-5-113 1471-2105-5-113 [pii].

Garrison, Erik, and Gabor Marth. 2012. "Haplotype-Based Variant Detection from Short-Read Sequencing," July. http://arxiv.org/abs/1207.3907.

Grattapaglia, D, and R Sederoff. 1994. "Genetic Linkage Maps of Eucalyptus Grandis and Eucalyptus Urophylla Using a Pseudo-Testcross: Mapping Strategy and RAPD Markers." *Genetics* 137 (4): 1121–37. http://www.ncbi.nlm.nih.gov/pubmed/7982566.

Haas, B J, A L Delcher, S M Mount, J R Wortman, R K Smith Jr., L I Hannick, R Maiti, et al. 2003. "Improving the Arabidopsis Genome Annotation Using Maximal Transcript Alignment Assemblies." *Nucleic Acids Res* 31 (19):5654–66. http://www.ncbi.nlm.nih.gov/pubmed/14500829 http://pubmedcentralcanada.ca/picrender.cgi?accid=PMC206470&blobtype=pdf.

Haas, Brian J, Steven L Salzberg, Wei Zhu, Mihaela Pertea, Jonathan E Allen, Joshua Orvis, Owen White, C Robin Buell, and Jennifer R Wortman. 2008. "Automated Eukaryotic Gene Structure Annotation Using EVidenceModeler and the Program to Assemble Spliced Alignments." *Genome Biology* 9 (1): R7. doi:10.1186/gb-2008-9-1-r7.

Hu H, Uesaka M, Guo S, Shimai K, Lu TM, Li F, Fujimoto S, Ishikawa M, Liu S, Sasagawa Y, Zhang G, Kuratani S, Yu JK, Kusakabe TG, Khaitovich P, Irie N; EXPANDE Consortium 2017. "Constrained vertebrate evolution by pleiotropic genes." *Nat Ecol Evol.* doi: 10.1038/s41559-017-0318-0. [Epub ahead of print] PubMed PMID: 28963548.

Huang, S, Z Chen, G Huang, T Yu, P Yang, J Li, Y Fu, S Yuan, S Chen, and A Xu. 2012. "HaploMerger: Reconstructing Allelic Relationships for Polymorphic Diploid Genome Assemblies." *Genome Res* 22 (8): 1581–88. doi:10.1101/gr.133652.111.

Huang S, Kang M, Xu A 2017. "HaploMerger2: rebuilding both haploid sub-assemblies from high-heterozygosity diploid genome assembly." *Bioinformatics* 33(16):2577-2579. doi: 10.1093/bioinformatics/btx220. PMID: 28407147.

Jaffe, D B, J Butler, S Gnerre, E Mauceli, K Lindblad-Toh, J P Mesirov, M C Zody, and E S Lander. 2003. "Whole-Genome Sequence Assembly for Mammalian Genomes: Arachne 2." *Genome Res* 13 (1): 91–96. doi:10.1101/gr.828403.

Li, H, and R Durbin. 2009. "Fast and Accurate Short Read Alignment with Burrows-Wheeler Transform." In *Bioinformatics*, 2009/05/20, 25:1754–60.. doi:10.1093/bioinformatics/btp324.

———. 2010. “Fast and Accurate Long-Read Alignment with Burrows-Wheeler Transform.” *Bioinformatics* 26 (5). Wellcome Trust Sanger Institute, Wellcome Genome Campus, Cambridge, CB10 1SA, UK.: 589–95. doi:btp698 [pii] 10.1093/bioinformatics/btp698.

Lieberman-Aiden, Erez, Nynke L van Berkum, Louise Williams, Maxim Imakaev, Tobias Ragoczy, Agnes Telling, Ido Amit, et al. 2009. “Comprehensive Mapping of Long-Range Interactions Reveals Folding Principles of the Human Genome.” *Science (New York, N.Y.)* 326 (5950): 289–93. doi:10.1126/science.1181369.

Ma, Ay, Lee, Gulsoy, Deng, Cook, Hesson, Cavanaugh, Ware, Krumm, Shendure, Blau, Disteche, Noble, Duan. 2014. Fine-scale chromatin interaction maps reveal the cis-regulatory landscape of human lincRNA genes. Nat. Methods, 12, pp. 71-78, 10.1038/nmeth.3205

Margarido, G R A, A P Souza, and A A F Garcia. 2007. “OneMap: Software for Genetic Mapping in Outcrossing Species.” *Hereditas* 144 (3): 78–79. doi:10.1111/j.2007.0018-0661.02000.x.

Nakatani, Y, H Takeda, Y Kohara, and S Morishita. 2007. “Reconstruction of the Vertebrate Ancestral Genome Reveals Dynamic Genome Reorganization in Early Vertebrates.” *Genome Res* 17 (9). Department of Computational Biology, Graduate School of Frontier Sciences, The University of Tokyo, Kashiwa 277-0882, Japan. nakatani@cb.k.u-tokyo.ac.jp: 1254–65. doi:10.1101/gr.6316407.

Price, Morgan N, Paramvir S Dehal, and Adam P Arkin. 2010. “FastTree 2–approximately Maximum-Likelihood Trees for Large Alignments.” *PloS One* 5 (3): e9490.

Putnam, N H, T Butts, D E Ferrier, R F Furlong, U Hellsten, T Kawashima, M Robinson-Rechavi, et al. 2008. “The Amphioxus Genome and the Evolution of the Chordate Karyotype.” *Nature* 453 (7198). Department of Energy Joint Genome Institute, Walnut Creek, California 94598, USA.: 1064–71. doi:nature06967 10.1038/nature06967.

Putnam, Nicholas H., Brendan L. O’Connell, Jonathan C. Stites, Brandon J. Rice, Marco Blanchette, Robert Calef, Christopher J. Troll, et al. 2016. “Chromosome-Scale Shotgun Assembly Using an in Vitro Method for Long-Range Linkage.” *Genome Research* 26 (3): 342–50. doi:10.1101/gr.193474.115.

Simakov, O, F Marletaz, S J Cho, E Edsinger-Gonzales, P Havlak, U Hellsten, D H Kuo, et al. 2013. “Insights into Bilaterian Evolution from Three Spiralian Genomes.” *Nature*. doi:10.1038/nature11696.

Simão FA, Waterhouse RM, Ioannidis P, Kriventseva EV, Zdobnov EM. BUSCO: assessing genome assembly and annotation completeness with single-copy orthologs. Bioinformatics. 2015 Oct 1;31(19):3210-2. doi: 10.1093/bioinformatics/btv351

Smith JJ and Keinath MC 2015. “The sea lamprey meiotic map improves resolution of ancient vertebrate genome duplications.” Genome Res. 2015 Aug;25(8):1081-90. doi: 10.1101/gr.184135.114. PMID: 26048246.

Smith JJ, Timoshevskaya N, Ye C, et al. 2018. “The sea lamprey germline genome provides insights into programmed genome rearrangement and vertebrate evolution.” Nat Genet. 2018 Feb;50(2):270-277. doi: 10.1038/s41588-017-0036-1. PMID: 29358652.

Slater, Guy S C, and Ewan Birney. 2005. “Automated Generation of Heuristics for Biological Sequence Comparison.” *BMC Bioinformatics* 6 (1): 31.

Stanke, M, O Keller, I Gunduz, A Hayes, S Waack, and B Morgenstern. 2006. “AUGUSTUS: Ab Initio Prediction of Alternative Transcripts.” *Nucleic Acids Res* 34 (Web Server issue): W435-9. doi:34/suppl_2/W435 [pii] 10.1093/nar/gkl200.

Wang S, Zhang J, Jiao W, Li J, Xun X, Sun Y, Guo X, Huan P, Dong B, Zhang L, Hu X, Sun X, Wang J, Zhao C, Wang Y, Wang D, Huang X, Wang R, Lv J, Li Y, Zhang Z, Liu B, Lu W, Hui Y, Liang J, Zhou Z, Hou R, Li X, Liu Y, Li H, Ning X, Lin Y, Zhao L, Xing Q, Dou J, Li Y, Mao J, Guo H, Dou H, Li T, Mu C, Jiang W, Fu Q, Fu X, Miao Y, Liu J, Yu Q, Li R, Liao H, Li X, Kong Y, Jiang Z, Chourrout D, Li R, Bao Z (2017). “Scallop genome provides insights into evolution of bilaterian karyotype and development.” *Nat Ecol Evol.* 1(5):120. doi: 10.1038/s41559-017-0120. PubMed PMID: 28812685.
